# *Torreya grandis* Seed Polyphenols Protect RAW264.7 Macrophages by Inhibiting Oxidative Stress and Inflammation

**DOI:** 10.1002/fsn3.70682

**Published:** 2025-07-29

**Authors:** Ran Liu, Baogang Zhou, Kundian Che, Wei Gao, Haoyuan Luo, Jialin Yang, Zhanjun Chen, Wenzhong Hu

**Affiliations:** ^1^ College of Life Science Zhuhai College of Science and Technology Zhuhai China; ^2^ College of Life Science Jilin University Changchun China

**Keywords:** anti‐apoptosis, antioxidant activity, free radical scavenging, inflammation inhibition, polyphenols, *Torreya grandis* seed

## Abstract

The seeds of *Torreya grandis* are rich in polyphenols, yet their chemical characteristics and biological activities require systematic elucidation. In this study, *T. grandis* seed polyphenols (TGSP) were prepared using ultrasound‐assisted extraction (70% ethanol, solid‐to‐liquid ratio of 1:40 g/mL, 210 W power, 55°C, 50 min) coupled with AB‐8 macroporous resin purification. The resulting TGSP were characterized by ultraviolet–visible (UV–Vis) spectroscopy, Fourier‐transform infrared (FT‐IR) spectroscopy, and liquid chromatography–tandem mass spectrometry (LC–MS/MS). Subsequently, their biological activities were systematically evaluated through in vitro chemical assays and in a cellular model. Structural analysis indicated that TGSP are abundant in gallic acid and catechins. TGSP exhibited selective scavenging activities against different free radicals, with half‐maximal inhibitory concentrations (IC_50_) for 2,2′‐azino‐bis(3‐ethylbenzothiazoline‐6‐sulfonic acid) radical cation (ABTS·+), 1,1‐diphenyl‐2‐picrylhydrazyl radical (DPPH·), and hydroxyl radical (·OH) being 0.194 ± 0.015, 0.301 ± 0.020, and 1.013 ± 0.018 mg/mL, respectively. For comparison, the IC_50_ values of vitamin C (VC) for ABTS·+ and DPPH· radicals were well below 0.1 mg/mL, and its IC_50_ for ·OH radicals was 0.108 ± 0.011 mg/mL. At the cellular level, TGSP effectively inhibited the production of nitric oxide (NO), tumor necrosis factor‐alpha (TNF‐α), and interleukin‐6 (IL‐6) in lipopolysaccharide (LPS)‐induced RAW264.7 cells. Furthermore, TGSP significantly counteracted hydrogen peroxide (H_2_O_2_)‐induced oxidative stress by reducing levels of reactive oxygen species (ROS) and malondialdehyde (MDA), restoring the activity of antioxidant enzymes such as superoxide dismutase (SOD), and suppressing caspase‐3/9‐mediated apoptosis. In conclusion, these findings demonstrate that *T. grandis* seed polyphenols exert significant cytoprotective effects through a multi‐target mechanism, including direct free radical scavenging, inhibition of inflammation, and attenuation of oxidative stress‐induced damage. This suggests their potential for development as functional food ingredients or natural pharmaceutical components.

## Graphical abstract

*Torreya grandis* seed polyphenols (TGSP), primarily containing gallic acid and epicatechin, exhibit selective free radical scavenging activity. TGSP pretreatment effectively inhibits LPS‐induced inflammatory responses by reducing TNF‐α, NO, and IL‐6 production. TGSP also alleviates H_2_O_2_‐induced oxidative stress by enhancing antioxidant enzyme activities and reducing cell apoptosis.

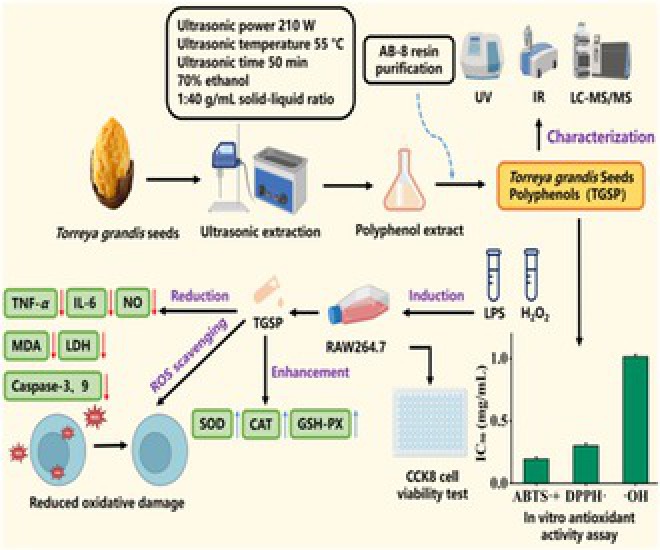

## 1 Introduction

*Torreya grandis*, a precious economic tree species endemic to China, is primarily distributed in regions such as Zhejiang, Anhui, Jiangsu, and Fujian (Suo et al. 2024; Yang et al. 2024). *T. grandis* seeds, the dried and mature seeds of the plant, possess significant nutritional and medicinal value (Gao et al. 2021; Suo et al. 2023, 2025). Recent studies have shown that *T. grandis* seeds are rich in various bioactive components, such as unsaturated fatty acids (especially sciadonic acid), volatile substances, and polyphenolic compounds (Lou et al. 2023; Yan et al. 2023; Zhou et al. 2019; Zongo et al. 2024). As plant secondary metabolites, polyphenols exhibit remarkable biological activities, including antioxidant and anti‐inflammatory effects (Dai and Mumper 2010; Jiang et al. 2024; Saini et al. 2024), suggesting that *T. grandis* seeds have broad application prospects in the development of functional foods and phytomedicines.

In recent years, research on *T. grandis* seed polyphenols (TGSP) has made significant progress. For instance, one study systematically identified as many as 107 flavonoids from *T. grandis* seeds and found that 19 of them were closely related to antioxidant activity (Liu et al. 2025). In addition to common phenolic acids and flavonoids reported in previous studies, such as protocatechuic acid and catechin, *T. grandis* seeds also contain a class of compounds characteristic of gymnosperms—biflavonoids, such as kayaflavone (Liu et al. 2025). Regarding its mechanism of action, the antioxidant effect of TGSP is thought to be primarily achieved by activating the Keap1‐Nrf2‐ARE signaling pathway (Gao et al. 2021). However, existing research has primarily focused on chemical composition analysis and preliminary in vitro antioxidant evaluations. The potential of TGSP to effectively inhibit cellular inflammation and exert comprehensive cytoprotective effects under oxidative stress—particularly regarding anti‐apoptosis—along with its underlying mechanisms, has not been systematically elucidated. This knowledge gap limits the comprehensive understanding of TGSP's bioactivities and hinders its application development.

Oxidative stress and inflammation are two closely intertwined pathophysiological processes (Widjaja et al. 2024), and their imbalance is considered a core driving factor in the development and progression of numerous chronic diseases, such as cardiovascular diseases, neurodegenerative diseases, and non‐alcoholic fatty liver disease (NAFLD) (Arroyave‐Ospina et al. 2021; Chaudhary et al. 2023; Leyane et al. 2022; Zhang and Tsao 2016). Macrophages play a pivotal role in these processes, serving as both a major source of ROS and a core effector cell in the inflammatory response (Kim et al. 2017). Therefore, identifying natural products that can simultaneously modulate both oxidative stress and inflammation in macrophages is of significant scientific importance for the prevention and treatment of related diseases.

Against this background, this study hypothesizes that TGSP possesses significant cytoprotective activity and can counteract oxidative and inflammatory damage through multi‐target mechanisms. To test this hypothesis, we first extracted and purified TGSP using an ultrasound‐assisted method and characterized its chemical composition. Subsequently, we employed the classic RAW264.7 macrophage model to establish an oxidative stress model induced by hydrogen peroxide (H_2_O_2_) and an inflammatory model induced by lipopolysaccharide (LPS). This study aims to systematically evaluate the efficacy and mechanisms of TGSP in anti‐inflammatory, antioxidant, and anti‐apoptotic activities, thereby providing a solid experimental basis for its further development and application.

## 2 Materials and Methods

### 2.1 Plant Material and Preparation

*T. grandis* seeds were collected from Shaoxing City, Zhejiang Province, China (29°42′02″–30°19′15″ N, 120°35′–120°46′39″ E) in 2024. The plant material was identified as the dried, mature seeds of *T. grandis* (family: Taxaceae) by Professor Wenzhong Hu. Prior to the experiments, the seeds were dehulled, pulverized using a high‐speed grinder, and sieved through a 20‐mesh screen to obtain a uniform powder. The resulting powder was stored at −20°C in the dark until use.

### 2.2 Reagents and Chemicals

Gallic acid (C_7_H_6_O_5_, 3,4,5‐trihydroxybenzoic acid) standard (purity ≥ 98%) was purchased from Shanghai Yuanye Bio‐Technology Co. Ltd. (Shanghai, China). Assay kits for 2,2‐diphenyl‐1‐picrylhydrazyl (DPPH·), 2,2′‐azino‐bis(3‐ethylbenzothiazoline‐6‐sulfonic acid) (ABTS·+), hydroxyl radical (·OH), and superoxide anion (O_2_·^−^) were procured from Nanjing Jiancheng Bioengineering Institute (Nanjing, China; Cat. No. A153‐1‐1, A015‐2‐1, A018‐1‐1, and A052‐1‐1, respectively). L‐Ascorbic acid (C_6_H_8_O_6_, (R)‐3,4‐dihydroxy‐5‐((S)‐1,2‐dihydroxyethyl)furan‐2(5H)‐one), also known as Vitamin C (purity ≥ 99%), was obtained from Macklin Biochemical Co. Ltd. (Shanghai, China). Ethanol anhydrous (C_2_H_5_OH), n‐hexane (C_6_H_14_), hydrochloric acid (HCl), and sodium hydroxide (NaOH) (all analytical grade) were from Sinopharm Chemical Reagent Co. Ltd. (Shanghai, China). AB‐8 macroporous adsorption resin (pore size: 13 nm; specific surface area: 480 m^2^/g) was supplied by Bona Agela Technologies Co. Ltd. (Tianjin, China).

Reagents for cell‐based assays included the following: The RAW264.7 murine macrophage cell line was obtained from the Cell Bank of the Chinese Academy of Sciences (Shanghai, China). LPS (purity ≥ 99%) was from Solarbio Science & Technology Co. Ltd. (Beijing, China). Dulbecco's Modified Eagle Medium (DMEM) high glucose, fetal bovine serum (FBS), trypsin, and penicillin–streptomycin solution (all cell‐culture grade) were purchased from Thermo Fisher Scientific (Waltham, MA, USA). The Cell Counting Kit‐8 (CCK‐8; Cat. No. C0037) was from Beyotime Biotechnology Co. Ltd. (Shanghai, China). Hydrogen peroxide (H_2_O_2_, 30%) and lysis buffer (cell‐culture grade) were from Sinopharm Chemical Reagent Co. Ltd. and Thermo Fisher Scientific, respectively. Enzyme‐linked immunosorbent assay (ELISA) kits for nitric oxide (NO; Cat. No. S0021S), tumor necrosis factor‐alpha (TNF‐α; Cat. No. PT512), and interleukin‐6 (IL‐6; Cat. No. PI326) were from Beyotime Biotechnology Co. Ltd. Assay kits for superoxide dismutase (SOD; Cat. No. A001‐3‐2), catalase (CAT; Cat. No. A007‐1‐1), glutathione peroxidase (GSH‐Px; Cat. No. A005‐1‐2), lactate dehydrogenase (LDH; Cat. No. A020‐1‐2), and malondialdehyde (MDA; Cat. No. A003‐1‐2) were from Nanjing Jiancheng Bioengineering Institute. DCFH‐DA, the Annexin V‐FITC/PI Apoptosis Detection Kit (Cat. No. C1383M), Caspase‐3 (Cat. No. C1116) and Caspase‐9 (Cat. No. C1158) ELISA kits, and the BCA Protein Assay Kit (Cat. No. P0012S) were all from Beyotime Biotechnology Co. Ltd.

Reagents for LC–MS/MS analysis were as follows: Methanol (CH₃OH), acetonitrile (CH₃CN), formic acid (HCOOH), acetic acid (CH₃COOH), and ammonium formate (NH₄COOH) (all LC–MS grade, purity ≥ 99.9%) were from Thermo Fisher Scientific. Ultrapure water (18.2 MΩ·cm) was prepared using a Milli‐Q Advantage A10 water purification system (Millipore, Billerica, MA, USA).

### 2.3 Instruments and Equipment

The main instruments and equipment used were as follows: a UV‐2600 UV–Visible Spectrophotometer (Shimadzu, Kyoto, Japan); a Multiskan SkyHigh Microplate Reader (Thermo Fisher Scientific, Waltham, MA, USA); an XH‐300A Ultrasonic Extractor (Beijing Xianghu Technology Development Co. Ltd., Beijing, China); an RE 5298A Rotary Evaporator (Shanghai Yarong Biochemical Instrument Factory, Shanghai, China); an IRPrestige‐21 Fourier‐Transform Infrared (FT‐IR) Spectrometer (Shimadzu, Kyoto, Japan); a Vanquish Ultra‐High‐Performance Liquid Chromatography (UHPLC) system coupled with a Q Exactive High‐Resolution Mass Spectrometer (Thermo Fisher Scientific, Waltham, MA, USA); an FA2204B Electronic Analytical Balance (Shanghai Precision & Scientific Instrument Co. Ltd., Shanghai, China); a 1580R High‐Speed Centrifuge (Beijing Aomijiaide Medical Technology Co. Ltd., Beijing, China); an HWS‐24 Constant Temperature Water Bath (Shanghai Yiheng Scientific Instrument Co. Ltd., Shanghai, China); a GZX‐9070MBE Blast Drying Oven (Shanghai Boxun Medical & Biological Instrument Corp., Shanghai, China); a BT100‐2J Peristaltic Pump (Baoding Lange Constant Flow Pump Co. Ltd., Baoding, China); a JC‐FW‐200 Pulverizer (Qingdao Juchuang Jiaheng Analytical Instrument Co. Ltd., Qingdao, China); a magnetic stirrer (Xi'an Bohui Instrument Co. Ltd., Xi'an, China); and a BIONOON‐VRF Vacuum Freeze Dryer (Shanghai Bionoon Biotechnology Co. Ltd., Shanghai, China).

Instruments for cell culture and analysis included an IX73 Inverted Fluorescence Microscope (Olympus, Tokyo, Japan); an HF240 CO_2_ Incubator (Heal Force Bio‐meditech Holdings Ltd., Shanghai, China); a BCM‐1000A Laminar Flow Clean Bench and a BSC‐1500IIA2‐X Biological Safety Cabinet (Sujing Group Antai Co. Ltd., Suzhou, China); a FACSCalibur Flow Cytometer (BD Biosciences, Franklin Lakes, NJ, USA); and an Epoch Microplate Reader (BioTek Instruments Inc., Winooski, VT, USA).

### 2.4 Pre‐Treatment of *T. grandis* Seeds

The *T. grandis* seed powder (300 g) was defatted by stirring with 3000 mL of n‐hexane (a 1:10 solid‐to‐liquid ratio, w/v) at 500 rpm for 90 min using a magnetic stirrer at room temperature (23°C ± 2°C) (Brglez Mojzer et al. 2016; Sridhar et al. 2021). After stirring, the residue was collected by filtration, and the defatting process was repeated two more times. The final defatted powder was then dried in a vacuum oven at 50°C for 3 h, sealed, and stored in the dark until further use.

### 2.5 Ultrasound‐Assisted Extraction (UAE) of TGSP

The extraction parameters were determined based on preliminary optimization using single‐factor experiments and Response Surface Methodology (RSM), the details of which are provided in Data S1. UAE was employed in this study due to its advantages, including high efficiency, mild operating conditions, and better preservation of bioactive compounds (Motikar et al. 2021). For practical convenience, the optimized process parameters were finalized as follows: an ultrasonic power of 210 W, a temperature of 55°C, a time of 50 min, a solid‐to‐liquid ratio of 1:40 g/mL, and 70% aqueous ethanol as the solvent.

The extraction was performed as follows: briefly, 5.0 g of the defatted *T. grandis* seed powder was accurately weighed and mixed with 200 mL of 70% ethanol solution (a 1:40 g/mL ratio). The mixture was then extracted in an XH‐300A ultrasonic extractor at 210 W and 55°C for 50 min. After extraction, the resulting liquid was filtered, and the filtrate was concentrated using a rotary evaporator at 45°C under a pressure of −0.08 MPa to remove the ethanol. Subsequently, the concentrate was freeze‐dried in a vacuum freeze dryer at −50°C to yield the crude TGSP extract. The crude extract was sealed and stored at −20°C in the dark.

The yield was calculated according to the following formula (Zheng et al. 2022):

$$\text{Yield}\;\left(\%\right)=\frac{\text{Weight of extract}\;\left(g\right)}{\text{Weight of initial sample}\;\left(g\right)}\times 100$$

### 2.6 Determination of Total Polyphenol Content

The total polyphenol content was determined using the Folin–Ciocalteu method (Tan et al. 2022). 100 mg of TGSP solid was accurately weighed and dissolved in 100 mL of 80% ethanol solution with ultrasonic assistance for 10 min (frequency 25 kHz, power 100 W) to obtain a sample solution with a concentration of 1 mg/mL. 0.5 mL of the sample solution was precisely pipetted and mixed with 3 mL of distilled water and 2.5 mL of 10% Folin–Ciocalteu reagent. After standing for 5 min, 2 mL of 7.5% Na_2_CO_3_ solution was added, followed by incubation in a 60°C water bath for 60 min and cooling to room temperature (23°C ± 2°C). The absorbance was measured at 765 nm using a 1 cm quartz cuvette, with gallic acid as the standard (concentration range 2–12 μg/mL). The linear regression equation was *y* = 87.214*x* + 0.0487, *R* ^2^ = 0.9991, and the results were expressed as gallic acid equivalents (mg GAE/g, DW). The total polyphenol content was determined using the Folin–Ciocalteu method. The calculation formulas were as follows (Tan et al. 2022):

$$\text{Total polyphenol content}\;\left(\%\right)=\frac{C\times V\times D}{\;W\;}\times 100\%$$

where: *C* is the polyphenol concentration in the sample solution calculated from the gallic acid standard curve (mg GAE/mL); *V* is the volume of the sample solution (mL); *D* is the dilution factor; *W* is the weight of the sample taken (mg, DW).

### 2.7 Purification of TGSP

TGSP was purified using AB‐8 macroporous adsorption resin chromatography (Ren et al. 2025). First, the AB‐8 resin was activated with 95% ethanol for 24 h, followed by washing with distilled water until colorless, and then wet‐packed into a column (inner diameter 1.6 cm, length 18.1 cm, packed with 25.7 g resin, bed height 15.3 cm). The crude *T. grandis* seed extract was dissolved in deionized water to a concentration of 1.5 mg/mL, filtered through a 0.45 μm membrane, and loaded onto the pre‐treated resin column at a flow rate of 2 mL/min, with a loading volume of 2 bed volumes (BV). After loading, the column was washed with 2 BV of deionized water at a flow rate of 2 mL/min to remove highly polar impurities. Subsequently, 75% ethanol was used as the eluent at a constant flow rate of 2 mL/min for 4 BV. The eluate was collected, concentrated under reduced pressure using a rotary evaporator at 45°C and −0.08 MPa to remove ethanol, and freeze‐dried in a vacuum freeze dryer at −50°C to obtain purified TGSP.

$$\text{Enrichment factor}=\frac{\text{Total polyphenol content of purified sample}\;\left(\%\right)}{\text{Total polyphenol content of crude sample}\;\left(\%\right)}$$

$$\text{Recovery rate}\;\left(\%\right)=\frac{\text{Total polyphenols after purification}}{\text{Total polyphenols before purification}}\times 100\%$$

### 2.8 UV–Vis Spectral Analysis of TGSP

Purified TGSP was dissolved in methanol and diluted to 0.1 mg/mL, filtered through a 0.22 μm membrane, and measured using a UV‐2600 UV–Vis spectrophotometer with a 1 cm quartz cuvette at room temperature (23°C ± 2°C). Full wavelength scanning was performed in the range of 200–400 nm with a scanning interval of 1 nm, using similarly treated methanol as a blank control (Tan et al. 2022).

### 2.9 FT‐IR Spectral Analysis of TGSP

TGSP was thoroughly ground and mixed with KBr powder (dried at 100°C for 8 h) at a ratio of 1:100 in an agate mortar and pressed into a transparent thin disc. The measurements were performed using an IRPrestige‐21 Fourier‐transform infrared spectrometer in an environment with relative humidity below 50%. Scanning was conducted in the range of 400–4000 cm^−1^ with a resolution of 4 cm^−1^, a scanning speed of 2 mm/s, and 40 scan accumulations (Tan et al. 2022).

### 2.10 LC–MS/MS Analysis of TGSP

Twenty milligrams of purified TGSP was accurately weighed and dissolved in 2 mL of 70% methanol, subjected to ultrasonic treatment (frequency 25 kHz, power 100 W) for 20 min, and filtered through a 0.22 μm PTFE membrane. Analysis was performed using a Vanquish ultra‐high‐performance liquid chromatography system coupled with a Q Exactive high‐resolution mass spectrometer. Chromatographic conditions: Waters ACQUITY UPLC HSS T3 column (2.1 × 100 mm, 1.8 μm), column temperature 40°C, mobile phase A was 0.1% formic acid aqueous solution, mobile phase B was 0.1% formic acid acetonitrile solution, flow rate 0.3 mL/min, gradient elution program: 0–1 min, 5% B; 1–10 min, 5%–20% B; 10–20 min, 20%–50% B; 20–25 min, 50%–95% B; 25–26 min, 95% B; 26–26.1 min, 95%–5% B; 26.1–30 min, 5% B. Mass spectrometry conditions: negative ion mode (ESI‐), m/z range 100–1000 Da, spray voltage 4.5 kV, desolvation temperature 500°C, desolvation gas flow rate 1000 L/h. Qualitative and relative quantitative analyses were performed using multiple reaction monitoring (MRM) mode (Zheng et al. 2022).

### 2.11 DPPH·Free Radical Scavenging Activity Assay

The DPPH· free radical detection kit from Nanjing Jiancheng Bioengineering Institute was used. Purified TGSP was prepared in 80% ethanol at different concentrations (0.1, 0.3, 0.5, 1.0, 2.0, 3.0, and 5.0 mg/mL). According to the kit instructions, 1.0 mL of sample solution was mixed with 1.0 mL of DPPH· working solution and allowed to react in the dark at room temperature (23°C ± 2°C) for 30 min, after which the absorbance was measured at 517 nm. An equal volume of 80% ethanol instead of the sample solution was used as the control, and an equal volume of 80% ethanol instead of DPPH· working solution was used as the sample blank. Vitamin C (0.1, 0.3, 0.5, 1.0, 2.0, 3.0, 5.0 mg/mL) was used as a positive control. The DPPH· free radical scavenging rate was calculated according to the following formula (Zheng et al. 2022), and the IC_50_ value of the sample was calculated through nonlinear regression analysis.

$$\text{Scavenging rate}\;\left(\%\right)=\left[1–\left(\frac{\text{Asample}-\text{Asample blank}}{\text{Acontrol}}\right)\right]\times 100\%$$

### 2.12 ABTS· + Free Radical Scavenging Activity Assay

The ABTS· + free radical detection kit from Nanjing Jiancheng Bioengineering Institute was used. Purified TGSP was prepared in 80% ethanol at different concentrations (0.1, 0.3, 0.5, 1.0, 2.0, 3.0, and 5.0 mg/mL). 0.5 mL of sample solution was mixed with 3.0 mL of ABTS· + working solution and allowed to react in the dark at room temperature (23°C ± 2°C) for 6 min, after which the absorbance was measured at 734 nm. An equal volume of 80% ethanol instead of the sample solution was used as the control, and an equal volume of 80% ethanol instead of ABTS· + working solution was used as the sample blank. Vitamin C (0.1, 0.3, 0.5, 1.0, 2.0, 3.0, and 5.0 mg/mL) was used as a positive control. The ABTS· + free radical scavenging rate and the IC_50_ value were determined as described in Section 2.11.

### 2.13 Hydroxyl Radical Scavenging Activity Assay

The hydroxyl radical (·OH) detection kit from Nanjing Jiancheng Bioengineering Institute was used. Purified TGSP was prepared in 80% ethanol at different concentrations (0.1, 0.3, 0.5, 1.0, 2.0, 3.0, and 5.0 mg/mL). According to the kit instructions, 0.2 mL of sample solution was mixed with 1.8 mL of hydroxyl radical reaction system and incubated at 37°C ± 1°C for 60 min, after which the absorbance was measured at 510 nm. An equal volume of 80% ethanol instead of the sample solution was used as the control, and an equal volume of 80% ethanol instead of hydroxyl radical reaction solution was used as the sample blank. Vitamin C (0.1, 0.3, 0.5, 1.0, 2.0, 3.0, and 5.0 mg/mL) was used as a positive control. The hydroxyl radical scavenging rate and the IC_50_ value were determined as described in Section 2.11.

### 2.14 Superoxide Anion Radical Scavenging Activity Assay

The superoxide anion radical (O_2_^−^·) detection kit from Nanjing Jiancheng Bioengineering Institute was used. Purified TGSP was prepared in 80% ethanol at different concentrations (0.1, 0.3, 0.5, 1.0, 2.0, 3.0, and 5.0 mg/mL). According to the kit instructions, 0.5 mL of sample solution was mixed with 2.0 mL of superoxide anion reaction system and incubated at 37°C ± 1°C for 30 min, after which the absorbance was measured at 560 nm. An equal volume of 80% ethanol instead of the sample solution was used as the control, and an equal volume of 80% ethanol instead of the superoxide anion reaction solution was used as the sample blank. Vitamin C (0.1, 0.3, 0.5, 1.0, 2.0, 3.0, and 5.0 mg/mL) was used as a positive control. The superoxide anion radical scavenging rate and the IC_50_ value were determined as described in Section 2.11.

### 2.15 Cell Culture

The RAW264.7 cell line was selected as the in vitro model for this study for several reasons. First, this cell line is a classic model for studying macrophage functions such as phagocytosis, inflammatory responses, and immunomodulation, and it exhibits a sensitive and stable response to inflammatory stimuli like LPS. Second, RAW264.7 cells are easy to culture and maintain, and they produce substantial amounts of inflammatory mediators (e.g., NO, TNF‐α, IL‐6, and ROS), making them an ideal tool for evaluating the anti‐inflammatory and antioxidant activities of natural products (Murray et al. 2014; Xie et al. 2020).

The RAW264.7 murine macrophage cells were cultured in Dulbecco's Modified Eagle Medium (DMEM) with high glucose, supplemented with 10% fetal bovine serum (FBS) and 1% penicillin–streptomycin. The cells were maintained in a humidified incubator at 37°C with a 5% CO_2_ atmosphere (Aki et al. 2020; Hu et al. 2022). They were subcultured every 2 days, and cells used for all experiments were kept within 15 passages to ensure the stability and reproducibility of the results.

### 2.16 Cell Viability Assay

The CCK‐8 method was used to evaluate the cytotoxicity of TGSP on RAW264.7 cells. Cells were seeded in 96‐well plates at a density of 5 × 10^3^ cells/well and cultured at 37°C under 5% CO_2_ for 24 h, followed by treatment with complete culture medium containing different concentrations of TGSP (0, 50, 100, 200, 400, 800 μg/mL) (final DMSO concentration < 0.1%), as DMSO at low concentrations (e.g., 0.1%) typically has no significant toxic effects on cells (Moskot et al. 2019; Sangweni et al. 2021; Święciło et al. 2024). The cells were further cultured for 24 h. Six replicate wells were set up for each concentration, and 10 μL of CCK‐8 reagent was added to each well, followed by incubation at 37°C for 2 h. The absorbance was measured at 450 nm using a microplate reader, with blank controls containing only culture medium and CCK‐8 reagent without cells (Chen et al. 2022; Ishikawa et al. 2016). The cell viability was calculated according to the following formula (Cai et al. 2019):

$$\text{Cell viability}\;\left(\%\right)=\frac{\text{Atreatment}-\text{Ablank}}{\text{Acontrol}-\text{Ablank}}\times 100\%$$

where Atreatment, Acontrol, and Ablank represent the absorbance values of the treatment group, control group (0 μg/mL TGSP), and blank group, respectively.

### 2.17 Establishment of the LPS‐Induced Inflammation Model

To establish the inflammation model, RAW264.7 cells were seeded in 24‐well plates at a density of 5 × 10^5^ cells/well. After 24 h of incubation, the cells were treated with various concentrations of LPS (0.5, 1, 2, 3, and 5 μg/mL) for another 24 h. Following treatment, the cell culture supernatants were collected to measure NO release using an NO assay kit based on the Griess reaction.

The principle of this assay is that the unstable NO molecule released by cells is rapidly oxidized to stable nitrite (NO_2_^−^) in the supernatant. Under acidic conditions, sulfanilamide in the Griess reagent reacts with NO_2_^−^ to form a diazonium salt. This salt then couples with N‐(1‐naphthyl) ethylenediamine to generate a pink azo compound. (Yan et al. 2024) The absorbance of this compound was measured at 540 nm, and the concentration of NO_2_^−^ was calculated by referencing a sodium nitrite standard curve, thereby indirectly reflecting the cellular NO release level (Kim et al. 2024). Each concentration was tested in triplicate. The optimal LPS concentration for subsequent experiments was determined based on the levels of NO production and changes in cell morphology.

### 2.18 Measurement of Inflammatory Mediators

RAW264.7 cells were seeded in 24‐well plates at a density of 5 × 10^5^ cells/well and cultured for 24 h. Subsequently, the cells were divided into the following groups: a control group (treated with complete medium only), an LPS group, and TGSP pretreatment groups. For the pretreatment groups, cells were pre‐incubated with various concentrations of TGSP (50, 100, 200, and 400 μg/mL) for 2 h. Then, the optimal concentration of LPS (as determined previously) was added to these wells and the LPS group wells, and the cells were co‐incubated for an additional 22 h. All treatments were performed in triplicate (Hyun et al. 2023).

At the end of the incubation period, the cell culture supernatants were collected. The concentration of NO was determined using the Griess assay. The concentrations of TNF‐α and IL‐6 were measured using their respective ELISA kits according to the manufacturer's protocols.

### 2.19 Establishment of H_2_O_2_‐Induced Oxidative Stress Model

To establish the oxidative stress model, RAW264.7 cells were seeded in 96‐well plates at a density of 5 × 10^3^ cells/well and cultured for 24 h. The cells were then treated with various concentrations of H_2_O_2_ (50, 100, 200, 400, 600, and 800 μmol/L) for 4 h. Following treatment, cell viability was assessed using the CCK‐8 assay. Each concentration was tested in sextuplicate wells (Shu et al. 2024). Based on the resulting dose–response curve, the concentration of H_2_O_2_ that reduced cell viability to approximately 50% was selected for subsequent experiments.

### 2.20 Measurement of Cellular Oxidative Stress Markers

#### 2.20.1 Experimental Design and Sample Preparation

The cells were divided into a control group (treated with complete medium only), an H_2_O_2_ model group, and TGSP pretreatment groups. For the treatment, cells were pre‐incubated with various concentrations of TGSP (50, 100, 200, and 400 μg/mL) for 2 h, followed by co‐incubation with the optimal concentration of H_2_O_2_ for 4 h. Each treatment was performed in triplicate.

After treatment, the cell culture supernatants were collected for the LDH release assay. For intracellular marker analysis, the adherent RAW264.7 cells were first washed with pre‐chilled phosphate‐buffered saline (PBS). Subsequently, cell lysis buffer containing protease inhibitors was added, and the cells were lysed on ice, followed by sonication to ensure complete disruption. The lysates were then centrifuged at 12,000× *g* for 15 min at 4°C, and the resulting supernatants (cell lysates) were collected for subsequent analysis (Wen et al. 2013; Zhang et al. 2019). All samples were stored at −80°C until use.

#### 2.20.2 Biochemical Assays

All assays for oxidative stress markers were performed using commercial kits from the Nanjing Jiancheng Bioengineering Institute, and all procedures strictly followed the manufacturer's instructions.

- SOD Activity Assay: The activity of SOD (Kit No. A001‐3‐2) was measured using the WST‐1 method. The principle of this assay is that superoxide anions (O_2_·^−^), generated by a xanthine oxidase system, reduce WST‐1 to a colored formazan product. SOD in the sample scavenges O_2_·^−^, thereby inhibiting formazan formation. The SOD activity was calculated by measuring the degree of inhibition via absorbance at 450 nm. The results are expressed as U/mg protein (Peskin and Winterbourn 2017).
- CAT Activity Assay: CAT activity (Kit No. A007‐1‐1) was determined by a spectrophotometric method. This assay is based on the characteristic absorbance of H_2_O_2_ at 240 nm. CAT present in the sample decomposes H_2_O_2_, and the enzyme activity is calculated by monitoring the rate of decrease in absorbance at 240 nm. The results are expressed as U/mg protein (Hadwan et al. 2024).
- GSH‐Px Activity Assay: GSH‐Px activity (Kit No. A005‐1‐2) was measured using a colorimetric method. The principle involves the GSH‐Px‐catalyzed oxidation of reduced glutathione (GSH) to oxidized glutathione (GSSG) by H_2_O_2_. The consumption rate of GSH (often coupled with the oxidation rate of NADPH, which is monitored at 340 nm) reflects the activity of GSH‐Px. The results are expressed as U/mg protein (Song et al. 2024).
- MDA Content Assay: The content of MDA (Kit No. A003‐1‐2), a major end product of lipid peroxidation, was determined using the thiobarbituric acid (TBA) method. Under acidic and high‐temperature conditions, MDA reacts with TBA to form a red trimethine complex with a maximum absorption peak at 532 nm. The MDA content was calculated from the absorbance of this substance and is expressed as nmol/mg protein (De Leon and Borges 2020).
- LDH Release Assay: The release of LDH (Kit No. A020‐1‐2) from damaged cells into the supernatant was measured to assess cell membrane integrity. LDH catalyzes the conversion of lactate to pyruvate, which concurrently reduces NAD^+^ to NADH. The rate of increase in NADH absorbance at 340 nm is proportional to LDH activity. The results are expressed as U/mg protein (Chan et al. 2013).

#### 2.20.3 Protein Quantification and Normalization

The total protein concentration in the cell lysates was determined using the bicinchoninic acid (BCA) method for the normalization of all intracellular markers. The principle of the BCA assay is that under alkaline conditions, protein reduces cupric ions (Cu^2+^) to cuprous ions (Cu^+^). These cuprous ions then form a stable, purple‐blue complex with the BCA reagent. This complex exhibits a maximum absorbance peak at 562 nm, and its absorbance is directly proportional to the protein concentration, allowing for accurate colorimetric quantification (Cortés‐Ríos et al. 2020).

### 2.21 Measurement of Intracellular ROS Levels

Intracellular ROS levels were measured using the ROS‐sensitive fluorescent probe 2′,7′‐dichlorodihydrofluorescein diacetate (DCFH‐DA). The principle of this assay is based on the ability of the non‐fluorescent DCFH‐DA to freely cross the cell membrane. Once inside the cell, it is hydrolyzed by intracellular esterases to 2′,7′‐dichlorodihydrofluorescein (DCFH). DCFH is then oxidized by ROS (such as H_2_O_2_ and ·OH) into the highly fluorescent compound 2′,7′‐dichlorofluorescein (DCF) (Kim and Xue 2020; Yu et al. 2021). Therefore, the fluorescence intensity of DCF quantitatively reflects the total intracellular ROS level.

#### 2.21.1 Fluorescence Microscopy Imaging

Cell grouping and treatments were performed as described in Section 2.20. After treatment, the culture medium was discarded, and the cells were gently washed twice with phosphate‐buffered saline (PBS). Subsequently, a DCFH‐DA working solution (final concentration: 10 μmol/L), diluted in serum‐free medium, was added to each well. The cells were then incubated in the dark for 30 min in a CO_2_ incubator at 37°C. Following incubation, the probe solution was discarded, and the cells were carefully washed three times with pre‐chilled PBS to completely remove any probe that had not entered the cells. After the final wash, an appropriate volume of PBS was added to each well. Fluorescence images were immediately captured using an IX73 inverted fluorescence microscope at an excitation wavelength of 488 nm and an emission wavelength of 525 nm (Kim and Xue 2020). For each group, at least five random fields of view were photographed to qualitatively assess the production of intracellular ROS.

#### 2.21.2 Flow Cytometry Analysis

For quantitative analysis, parallel plates of cells were prepared. Following the probe incubation and washing steps, the cells were detached by adding an appropriate volume of 0.25% trypsin–EDTA solution and incubating at 37°C for 1–2 min. Once the cells became rounded and the intercellular spaces increased, the digestion was terminated by adding complete medium containing 10% FBS. The cells were gently pipetted to form a single‐cell suspension and then transferred to flow cytometry tubes. The cell suspension was centrifuged at 500× *g* for 5 min at 4°C. The supernatant was discarded, and the cell pellet was resuspended in 400 μL of pre‐chilled PBS. The samples were immediately analyzed using a FACSCalibur flow cytometer. The signal was acquired through the FITC channel (corresponding to green fluorescence), and at least 10,000 cell events were recorded for each sample (Fong et al. 2017).

#### 2.21.3 Data Analysis

The experimental data were analyzed using FlowJo software (v10.9.0, BD Life Sciences, Ashland, OR, USA) and ImageJ software (v1.54p, U.S. National Institutes of Health, Bethesda, MD, USA). The mean fluorescence intensity (MFI) was calculated to quantify the average level of intracellular ROS. Each group was measured in triplicate.

### 2.22 Cell Apoptosis Analysis

#### 2.22.1 Annexin V‐FITC/PI Staining by Flow Cytometry

Cell treatments were performed as described in Section 2.20. After the incubation period, cells were collected and washed twice with PBS. Staining was then performed using an Annexin V‐FITC/PI dual staining kit according to the manufacturer's instructions. Briefly, the cell pellet was resuspended in 100 μL of binding buffer, to which 5 μL of Annexin V‐FITC and 5 μL of PI were added. After incubating at room temperature in the dark for 15 min, 400 μL of binding buffer was added to the suspension. The rate of apoptosis was immediately analyzed by flow cytometry, and the data were processed using FlowJo software (v10.9.0, BD Life Sciences, Ashland, OR, USA).

#### 2.22.2 Caspase‐3 and Caspase‐9 Activity Assays

Concurrently, the activities of caspase‐3 and caspase‐9 were determined using their respective ELISA kits. Following the treatments, cells were collected, and cell lysates were prepared according to the kit manufacturer's instructions. The assay was performed based on the double‐antibody sandwich ELISA principle. Samples and standards were added to microplate wells pre‐coated with a specific antibody. After incubation and washing, an enzyme‐labeled antibody was added. Following a second incubation and wash step, a substrate solution was added for color development. The absorbance was measured at 450 nm using a microplate reader. The concentrations of Caspase‐3 and Caspase‐9 were calculated from their respective standard curves and expressed as U/mg protein.

### 2.23 Statistical Analysis

All experiments were independently repeated three times, and the results are expressed as the mean ± standard deviation (SD). The normality of the data distribution was assessed using the Shapiro–Wilk test. For data that followed a normal distribution, the homogeneity of variances was first evaluated using Bartlett's test. Subsequently, one‐way analysis of variance (ANOVA) was performed, followed by Duncan's multiple range test for post hoc comparisons between groups. For data that were not normally distributed or had unequal variances, the Kruskal‐Wallis nonparametric test was used, followed by Dunn's post hoc test for multiple comparisons. A *p*‐value of < 0.05 was considered to indicate statistical significance. Statistical analyses were performed using SPSS software (version 25.0).

Statistical differences between treatment groups are indicated in the figures by different letters (e.g., a, b, c). All graphs were created using OriginPro 2021 software (OriginLab Corporation, Northampton, MA, USA).

## 3 Results and Discussion

### 3.1 Purification Results of TGSP

AB‐8 macroporous resin was used to purify the TGSP extract, increasing the total polyphenol content from 15.89% ± 0.42% to 76.37% ± 0.32%, achieving an enrichment factor of 4.8‐fold. The recovery rate of the purification process was 82.65% ± 1.57%, with a total yield of 1.88% ± 0.13% (based on the DW of raw material). The purified sample appeared as a brownish powder, readily soluble in water and low‐concentration ethanol, providing quality assurance for subsequent structural characterization and biological activity studies.

### 3.2 UV–Vis and FT‐IR Spectral Analysis of TGSP

**FIGURE 1:**
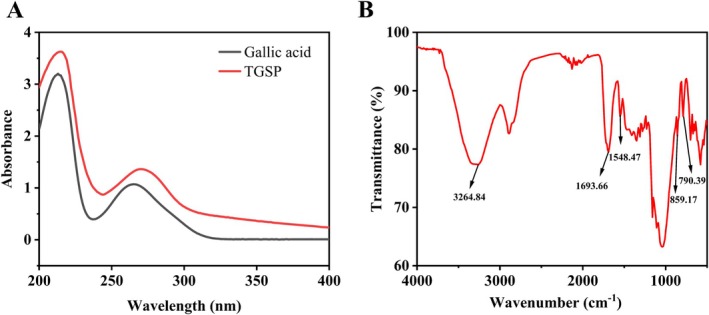
UV–Vis (A) and FT‐IR (B) spectra of TGSP.

The results of the UV–Vis and FT‐IR spectral analyses of TGSP are presented in Figure 1. The UV–Vis analysis revealed that TGSP exhibited a distinct characteristic absorption peak at 270 nm (Figure 1A), which was highly consistent with the absorption profile of the gallic acid standard. This peak is attributed to the π‐π* electron transitions within the aromatic rings of the polyphenolic compounds. The maximum absorbance of TGSP was slightly higher than that of gallic acid, suggesting that TGSP contains more complex polyphenolic structures.

The FT‐IR spectrum of TGSP (Figure 1B) displayed a broad and strong absorption band at 3264.84 cm^−1^, characteristic of the stretching vibrations of abundant hydroxyl (‐OH) groups in the polyphenol molecules (Pan et al. 2003). A significant absorption peak at 1693.66 cm^−1^ corresponded to the stretching vibration of ester carbonyl (C=O) groups (Jia et al. 2017), indicating the possible presence of gallate ester structures in the sample (Wongsa et al. 2022). The medium‐intensity absorption band at 1548.47 cm^−1^ was attributed to the skeletal vibration of the aromatic ring, further confirming the aromatic nature of the sample (Riaz et al. 2023). In the fingerprint region, characteristic peaks at 839.17 and 790.39/cm represented the C‐H out‐of‐plane bending vibrations of ortho‐ and para‐substituted aromatic rings, respectively (Fu et al. 2021), suggesting that TGSP contains aromatic structures with different substitution patterns (Tamer et al. 2023).

Collectively, the UV–Vis and FT‐IR analyses confirm that TGSP is rich in polyphenolic structures. Its functional group characteristics, such as abundant phenolic hydroxyls, ester carbonyl groups, and aromatic rings with various substitution patterns, are highly consistent with the chemical structures of phenolic substances like gallic acid and catechins.

### 3.3 LC–MS/MS Analysis of TGSP Composition

**FIGURE 2:**
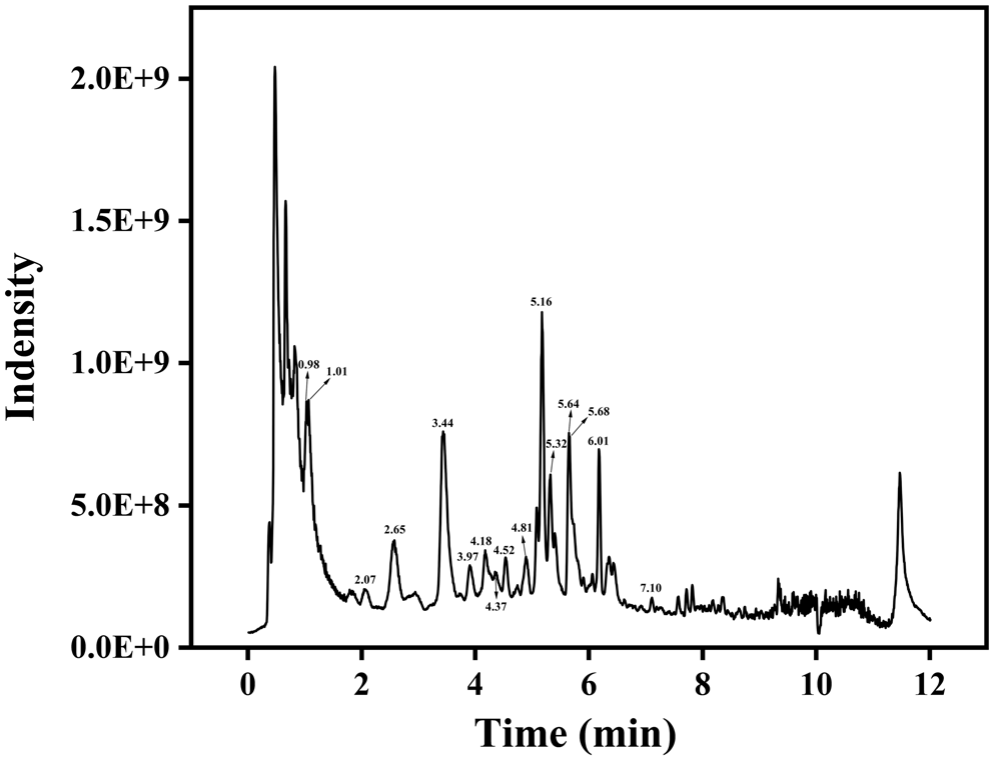
Total ion chromatogram of TGSP in ESI negative ion mode LC–MS/MS.

LC–MS/MS analysis identified more than 10 polyphenolic compounds in TGSP (Table 1). Among these, gallic acid (18,935.4 ng/mL) and epicatechin (4368.81 ng/mL) were the most abundant components. Other major polyphenolic compounds included catechin (4293.20 ng/mL), 3,4‐dihydroxybenzoic acid (2606.73 ng/mL), and 4‐hydroxybenzoic acid (1550.75 ng/mL). Figure 2 shows the total ion chromatogram (TIC) of the TGSP components obtained in the negative electrospray ionization (ESI^−^) mode. The high abundance of gallic acid and catechin derivatives in TGSP is strongly associated with its potent antioxidant activity (Bernatoniene and Kopustinskiene 2018; Chung et al. 2010; Kruk et al. 2022; Senanayake 2013).

**TABLE 1:** LC–MS/MS analysis results of TGSP.

Compound	Chemical formula	Ion mode	Molecular weight (g/mol)	Quantitative ion pair (Da)	Retention time (min)	Content (ng/mL)
Gallic acid	C_7_H_6_O_5_	ESI—	170.12	169.014/125.024	0.98	18,935.4
Phenylalanine	C_9_H_11_NO_2_	ESI—	165.19	164.07/147.04	1.01	269.41
3,4‐Dihydroxybenzoic acid	C_7_H_6_O_4_	ESI—	154.12	153.019/109.029	2.07	2606.73
Protocatechuic aldehyde	C_7_H_6_O_3_	ESI—	138.12	137.024/108.021	2.65	63.77
4‐Hydroxybenzoic acid	C_7_H_6_O_3_	ESI—	138.12	137.024/93.034	3.44	1550.75
Catechin	C_15_H_14_O_6_	ESI—	290.27	289.072/245.082	3.97	4293.20
Epicatechin	C_15_H_14_O_6_	ESI—	290.27	289.072/245.082	4.81	4368.81
Vanillic acid	C_8_H_8_O_4_	ESI—	168.14	167.035/152.011	4.18	685.80
Caffeic acid	C_9_H_8_O_4_	ESI—	180.15	179.035/135.045	4.37	540.26
Syringic acid	C_9_H_10_O_5_	ESI—	194.18	193.050/178.026	4.52	140.99
Vanillin	C_8_H_8_O_3_	ESI—	152.15	151.040/136.016	5.16	86.93
p‐Hydroxycinnamic acid	C_9_H_8_O_3_	ESI—	164.15	163.040/119.050	5.32	1285.54
trans‐Ferulic acid	C_10_H_10_O_4_	ESI—	194.18	193.050/134.037	5.64	1161.04
Sinapic acid	C_11_H_12_O_5_	ESI—	224.21	223.061/208.037	5.68	299.23
Benzoic acid	C_7_H_6_O_2_	ESI—	122.12	121.029/77.039	6.01	705.92
Hydrocinnamic acid	C_9_H_10_O_2_	ESI—	150.17	149.060/105.03	7.10	33.77

### 3.4 In Vitro Antioxidant Activity of TGSP

**FIGURE 3:**
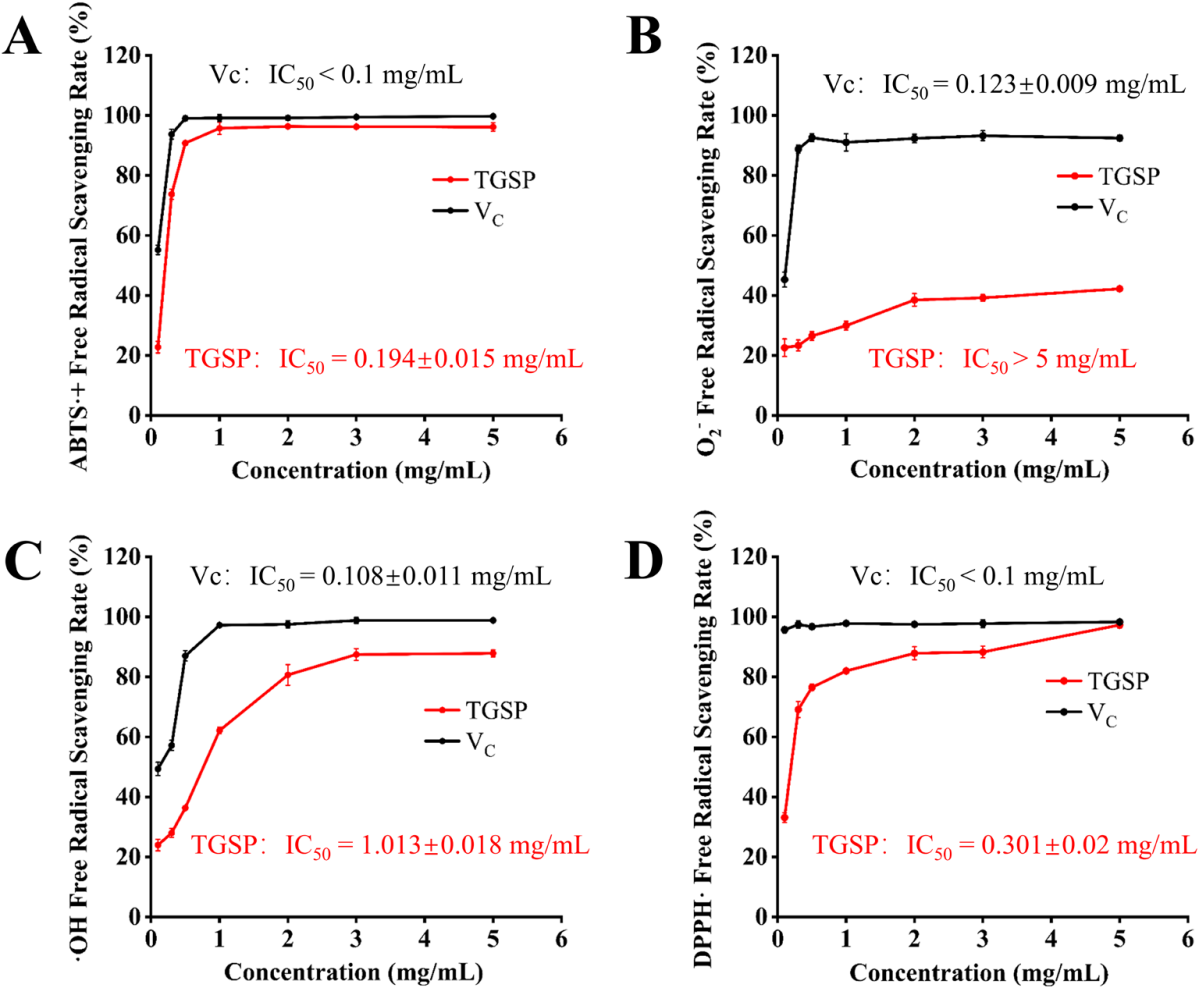
Scavenging activity of TGSP against different types of free radicals. (A) ABTS + radical scavenging activity; (B) O_2_^−^· superoxide anion radical scavenging activity; (C) OH hydroxyl radical scavenging activity; and (D) DPPH· radical scavenging activity.

In this study, the free radical scavenging activity of TGSP was systematically evaluated against four types of radicals—ABTS·+, DPPH·, ·OH, and O_2_·^−^—with vitamin C (VC) serving as a positive control. As shown in Figure 3, TGSP exhibited a dose‐dependent scavenging effect on all tested radicals. The order of its potency was as follows: ABTS·+ > DPPH· > ·OH > O_2_·^−^.

Specifically, the half‐maximal inhibitory concentration (IC_50_) of TGSP for ABTS· + was 0.194 ± 0.015 mg/mL, for DPPH· was 0.301 ± 0.020 mg/mL, and for ·OH was 1.013 ± 0.018 mg/mL. In contrast, TGSP demonstrated the weakest scavenging ability against the superoxide anion radical (O_2_·^−^). At the highest tested concentration (5.0 mg/mL), its scavenging rate was only 42.23% ± 0.85%, which did not reach 50% inhibition. Therefore, its IC_50_ value could not be determined in this assay.

As the positive control, pure VC exhibited significantly higher potency than TGSP in all assays. Its IC_50_ values for ABTS· + and DPPH· were both well below 0.1 mg/mL, while the values for ·OH and O_2_·^−^ were 0.108 ± 0.011 mg/mL and 0.123 ± 0.009 mg/mL, respectively. This result clearly indicates that although TGSP, as a crude extract, is less potent than pure VC, it still possesses significant and selective free radical scavenging capabilities, confirming its potential for development as a natural antioxidant.

Epicatechin and catechin, the main active components in TGSP, contain multiple ortho‐hydroxyl groups in their molecular structures, which can effectively capture free radicals and form relatively stable semiquinone radicals (Foss et al. 2022). This explains the potent scavenging ability of TGSP against ABTS· + and DPPH· radicals. The differential scavenging activity of TGSP against various radicals is likely related to the different chemical properties and reaction mechanisms of the radicals themselves (Yan et al. 2016).

### 3.5 Effects of TGSP on RAW264.7 Cell Viability

**FIGURE 4:**
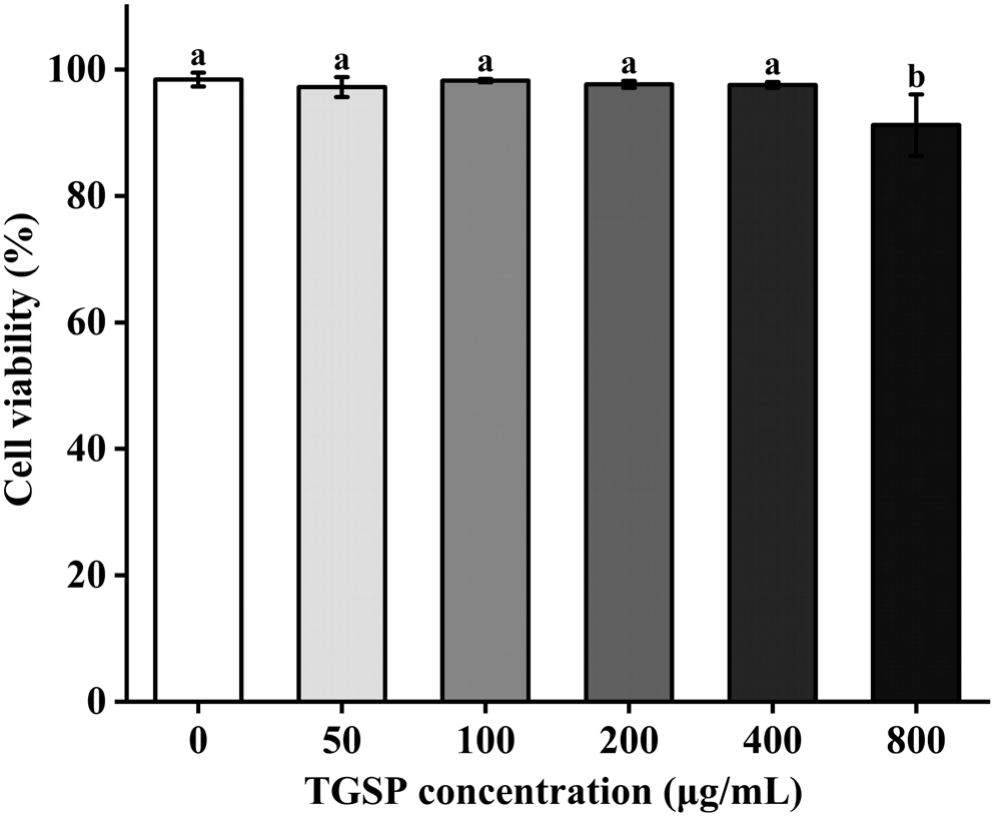
Effects of TGSP on RAW264.7 cell viability. Values are presented as means ± SD of three independent experiments. Bars with different letters (a, b) indicate significant differences (*p* < 0.05).

TGSP exhibited no significant effect on the viability of RAW264.7 cells at concentrations of 400 μg/mL and below (cell viability > 96%; *p* = 0.350, 0.805, 0.362, and 0.310, respectively, all > 0.05). In contrast, the cell viability in the group treated with 800 μg/mL of TGSP significantly decreased to 91.23% ± 4.85% (*p* = 0.014) compared to the control group's viability of 98.44% ± 1.01% (Figure 4). Based on these cytotoxicity assessment results, TGSP concentrations of ≤ 400 μg/mL were selected for subsequent experiments.

This result indicates that TGSP has good biocompatibility with the RAW264.7 macrophage cell line within a lower concentration range (< 400 μg/mL), providing a safe dose range for subsequent anti‐inflammatory and immunomodulatory studies. Although a statistically significant difference was observed at the 800 μg/mL concentration, the cell viability remained above 90%, suggesting that TGSP exhibits only mild cytotoxicity even at high concentrations. The selection of ≤ 400 μg/mL for subsequent experiments aligns with the consideration of a safety margin in pharmacological research, ensuring that the observed biological effects originate from the specific activity of TGSP rather than from non‐specific cell damage (Bognar et al. 2013).

### 3.6 Determination of Optimal Working Concentrations for Cell Models

**FIGURE 5:**
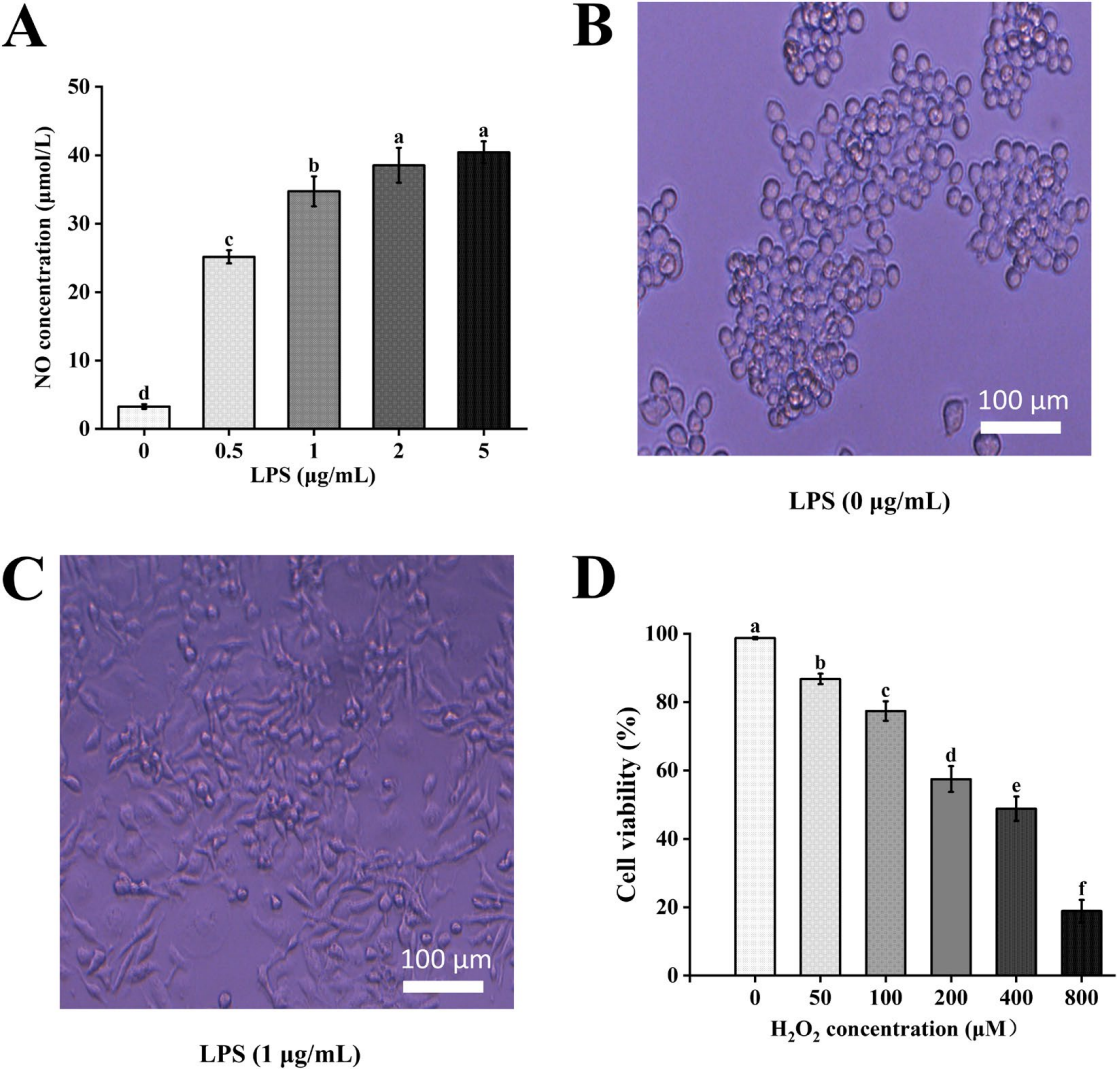
Optimization of working concentrations for cell models. (A) Dose‐dependent stimulation of NO release by LPS. (B, C) Morphological transition of RAW264.7 cells from a resting (B) to an activated state (C) upon stimulation with 1 μg/mL LPS. (D) Dose‐dependent reduction in cell viability by H_2_O_2_. Values are presented as means ± SD of three independent experiments. Bars with different letters (a, b) indicate significant differences (*p* < 0.05).

Prior to subsequent pharmacodynamic evaluations, we first optimized the establishment conditions for the inflammation and oxidative stress damage models. To determine the optimal concentration for the LPS‐induced inflammation model, we measured the amount of NO released from RAW264.7 cells after treatment with various concentrations of LPS. The results, shown in Figure 5A, indicated that LPS significantly stimulated NO production in a dose‐dependent manner. The NO concentrations corresponding to the 0–5 μg/mL treatment groups were 3.277 ± 0.339, 25.162 ± 0.956, 34.731 ± 2.190, 38.537 ± 2.55, and 40.446 ± 1.578 μmol/L, respectively (*p*‐values: 0.00017, 0.0013, 0.0015, and 0.00036, all < 0.05). This effect reached a desirable level and approached a plateau at 1 μg/mL (*p* = 0.0013). The morphological transition from a round, resting state (Figure 5B) to an activated and polarized state with extended pseudopods (Figure 5C) also visually confirmed that 1 μg/mL of LPS successfully activated the macrophage inflammatory pathway.

Subsequently, the H_2_O_2_ concentration for establishing the oxidative stress damage model was screened using the CCK‐8 assay. As shown in Figure 5D, the cytotoxic effect of H_2_O_2_ was also significantly dose‐dependent. The cell viabilities corresponding to the 0–800 μmol/L treatment groups were 98.746% ± 0.337%, 86.800% ± 1.508%, 77.414% ± 2.856%, 57.523% ± 3.763%, 48.836% ± 3.554%, and 18.882% ± 3.279%, respectively (*p*‐values: 0.0038, 0.0054, 0.0026, 0.0016, and 0.0005, all *p* < 0.05). At a concentration of 400 μmol/L, the cell viability was reduced to approximately 50% (48.8% ± 3.6%, *p* = 0.0016).

In summary, the final concentrations selected for subsequent experiments were 1 μg/mL for LPS stimulation and 400 μmol/L for H_2_O_2_‐induced damage.

### 3.7 TGSP Inhibits LPS‐Induced Inflammatory Response in RAW264.7 Cells

**FIGURE 6:**
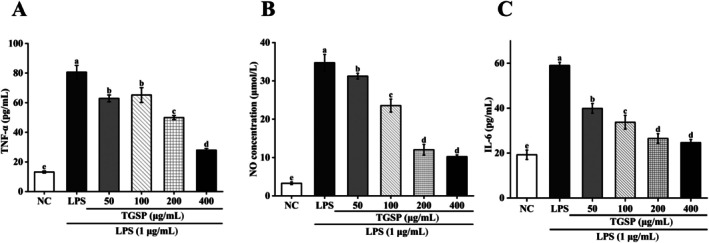
TGSP inhibits LPS‐induced inflammatory cytokine production in RAW264.7 cells. (A) TNF‐*α* levels; (B) NO production; and (C) IL‐6 release. Values are presented as means ± SD of three independent experiments. Bars with different letters (a, b) indicate significant differences (*p* < 0.05).

Compared to the negative control (NC) group, stimulation with LPS significantly increased the production of various inflammatory mediators in RAW264.7 cells. Specifically, the levels of the inflammatory cytokine TNF‐α (*p* = 0.0012), NO (*p* = 0.0013), and IL‐6 (*p* = 0.0000296) were all statistically significantly elevated after LPS treatment. However, pretreatment with TGSP effectively inhibited this process. At the highest concentration (400 μg/mL), TGSP markedly reduced the LPS‐induced TNF‐α level from 80.64 ± 4.57 pg/mL to 27.95 ± 1.12 pg/mL (*p* = 0.0015), the NO level from 34.73 ± 2.19 μmol/L to 10.23 ± 0.45 μmol/L (*p* = 0.0019), and the IL‐6 level from 58.99 ± 1.45 pg/mL to 24.64 ± 1.44 pg/mL (*p* = 0.000008277) (Figure 6), demonstrating significant anti‐inflammatory activity.

The inhibitory effect of TGSP on LPS‐induced inflammatory responses reveals its potential as a multifunctional bioactive component. LPS, a major component of the outer membrane of Gram‐negative bacteria, activates macrophages via Toll‐like receptor 4 (TLR4), triggering a series of inflammatory cascades (Wei et al. 2024). In this study, we confirmed that TGSP dose‐dependently and effectively inhibited the production of TNF‐α, NO, and IL‐6 in LPS‐stimulated RAW264.7 cells. Within the signal transduction network of inflammation, the nuclear transcription factor NF‐κB is considered a central hub that regulates the expression of numerous pro‐inflammatory genes, including those encoding TNF‐α, IL‐6, and iNOS (Lawrence 2009). Concurrently, the MAPK family (including p38, JNK, and ERK), acting as key upstream kinases (Jagodzik et al. 2018), also plays a crucial regulatory role in the production of these inflammatory mediators upon activation by LPS (Ren et al. 2020). Therefore, based on the potent anti‐inflammatory effects of TGSP we observed, it is reasonable to speculate that its mechanism of action may be associated with the inhibition of LPS‐induced activation of the NF‐κB and/or MAPK signaling pathways, although this requires further experimental verification.

### 3.8 Protective Effects of TGSP Against H_2_O_2_‐Induced Oxidative Stress in RAW264.7 Cells

H_2_O_2_, a key ROS, can penetrate cell membranes and generate highly reactive hydroxyl radicals intracellularly, leading to lipid peroxidation, protein modification, and DNA damage (Valverde et al. 2018). Oxidative stress is a critical trigger in the development and progression of many common diseases (Chandra Dash et al. 2025; Muscolo et al. 2024).

**FIGURE 7:**
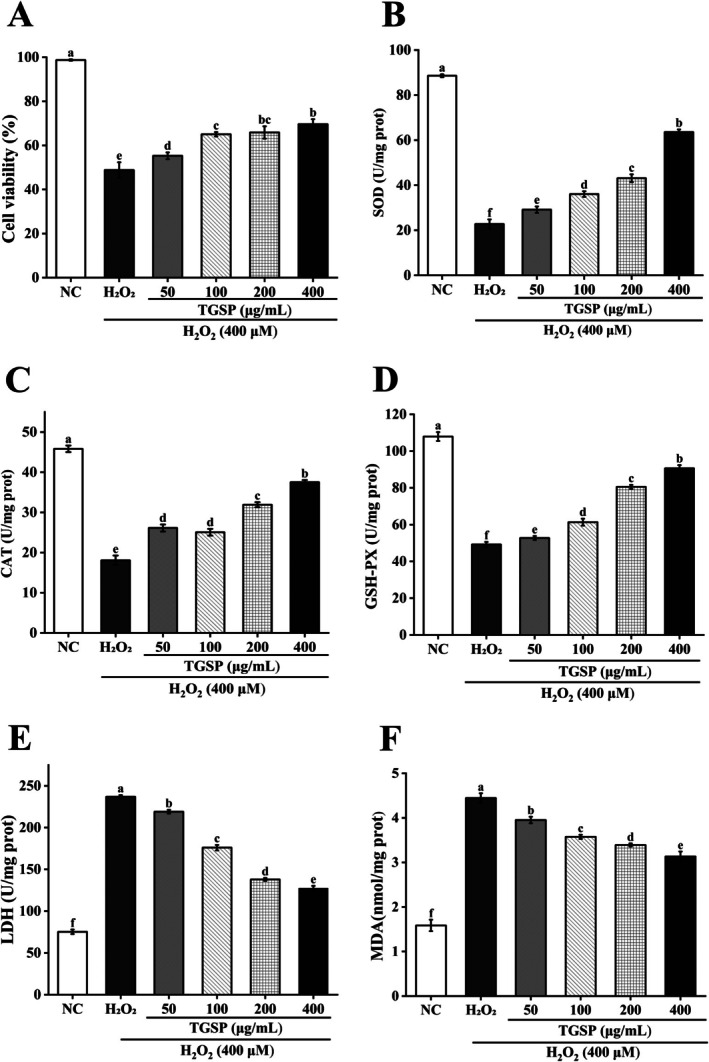
Protective effects of TGSP against H_2_O_2_‐induced oxidative stress in RAW264.7 cells. (A) Cell viability; (B) SOD activity; (C) CAT activity; (D) GSH‐Px activity; (E) LDH release; (F) MDA content. Values are presented as means ± SD of three independent experiments. Bars with different letters (a, b) indicate significant differences (*p* < 0.05).

Treatment with H_2_O_2_ (400 μM) significantly reduced the viability of RAW264.7 cells to 49.13% ± 3.55% (*p* = 0.0016). However, pretreatment with TGSP effectively mitigated this oxidative damage in a dose‐dependent manner. Compared to the damage model group treated with H_2_O_2_ alone, pretreatment with TGSP at concentrations of 50, 100, 200, and 400 μg/mL significantly increased cell viability to 57.11% ± 1.56%, 65.08% ± 0.98%, 65.92% ± 2.78%, and 69.64% ± 2.24%, respectively (Figure 7A; *p*‐values: 0.07, 0.011, 0.0034, and 0.0021).

As expected, exposure to H_2_O_2_ alone, compared to the normal control group, significantly inhibited intracellular antioxidant enzyme activities and increased the levels of oxidative damage markers (*p* < 0.05). Specifically, H_2_O_2_ treatment reduced SOD activity to 22.77 ± 2.05 U/mg protein (*p* = 0.000095), CAT activity to 18.09 ± 1.17 U/mg protein (*p* = 0.000013), and GSH‐Px activity to 49.20 ± 1.38 U/mg protein (*p* = 0.000029). Concurrently, it increased MDA content to 4.44 ± 0.11 nmol/mg protein (*p* = 0.000011) and LDH release to 236.99 ± 1.90 U/mg protein (*p* = 0.00000043).

However, pretreatment with TGSP effectively reversed these H_2_O_2_‐induced changes in a dose‐dependent manner. At the highest concentration (400 μg/mL), TGSP pretreatment significantly restored SOD activity from 22.77 U/mg protein to 63.61 ± 1.13 U/mg protein (Figure 7B, *p* = 0.000062), CAT activity from 18.09 U/mg protein to 37.53 ± 0.54 U/mg protein (Figure 7C, *p* = 0.00019), and GSH‐Px activity from 49.20 U/mg protein to 90.63 ± 1.73 U/mg protein (Figure 7D, *p* = 0.0000085). Furthermore, TGSP treatment at this concentration significantly reduced LDH release from 236.99 U/mg protein to 127.48 ± 2.74 U/mg protein (Figure 7E, *p* = 0.0000022) and decreased MDA content from 4.44 nmol/mg protein to 3.13 ± 0.11 nmol/mg protein (Figure 7F, *p* = 0.00012).

Pretreatment with TGSP significantly increased the activities of key antioxidant enzymes such as SOD, CAT, and GSH‐Px in RAW264.7 cells (*p* < 0.05). These enzymes play a crucial role in scavenging peroxides and maintaining cellular redox balance (Samanta et al. 2024). TGSP exhibited a protective effect against H_2_O_2_‐induced oxidative stress damage, an effect primarily achieved by enhancing the endogenous antioxidant defense system (Chandimali et al. 2025; Mladenov et al. 2023; Xu et al. 2019).

### 3.9 TGSP Reduces H_2_O_2_‐Induced ROS Production and Cell Apoptosis

#### 3.9.1 TGSP Scavenges Intracellular ROS

**FIGURE 8:**
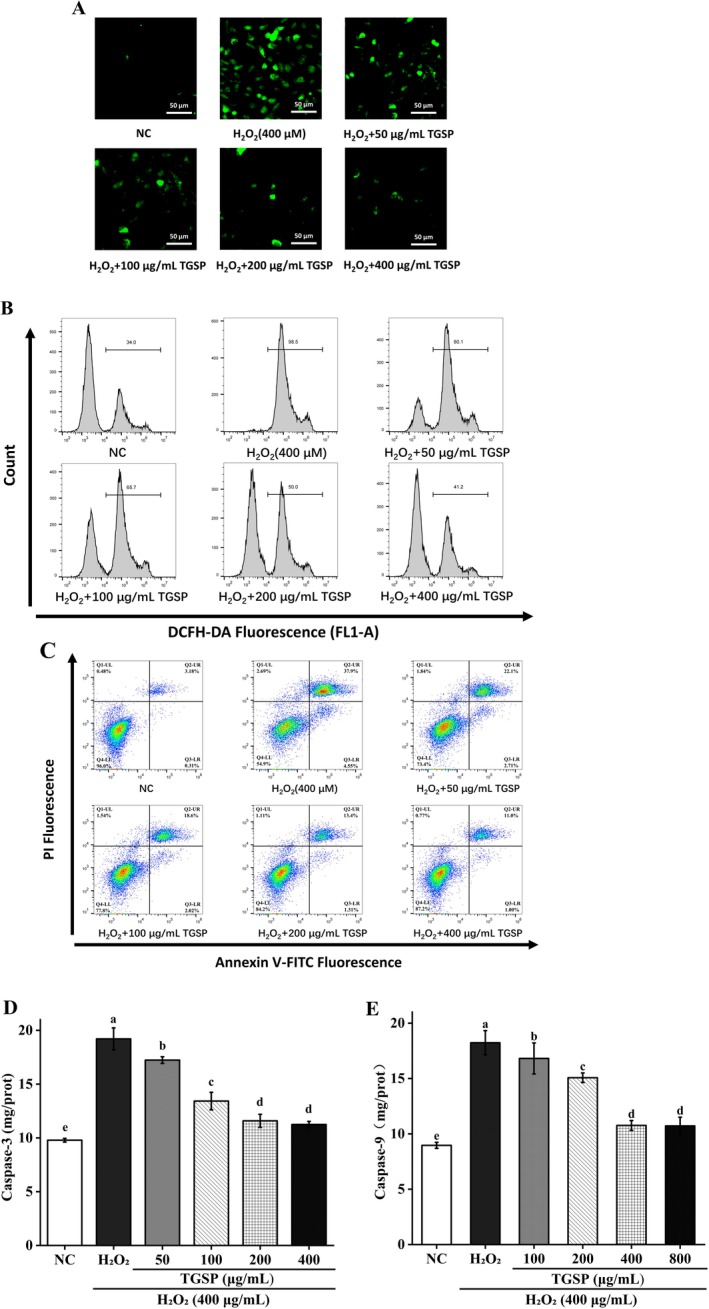
TGSP reduces H_2_O_2_‐induced ROS production and cell apoptosis in RAW264.7 cells. (A) DCFH‐DA fluorescence microscopy observation of ROS levels; (B) flow cytometry analysis of ROS production; (C) Annexin V‐FITC/PI double staining for cell apoptosis detection; (D) caspase‐3 activity; and (E) caspase‐9 activity. Values are presented as means ± SD of three independent experiments. Bars with different letters (a, b) indicate significant differences (*p* < 0.05).

DCFH‐DA fluorescence staining results showed that, compared to the normal control group, treatment with H_2_O_2_ (400 μM) significantly increased the intracellular ROS levels in RAW264.7 cells (*p* < 0.05). Quantitative analysis indicated that the mean fluorescence intensity of the cells dramatically rose from 28.83 ± 1.43 in the untreated group to 203.89 ± 5.87 in the H_2_O_2_ model group (*p* = 0.00019). However, pretreatment with TGSP effectively inhibited ROS production in a dose‐dependent manner (Figure 8A). As the TGSP concentration increased from 50 μg/mL to 400 μg/mL, the mean fluorescence intensity was significantly reduced to 146.57 ± 3.42, 113.82 ± 8.67, 74.48 ± 3.40, and 53.32 ± 4.00, respectively, compared to the H_2_O_2_ model group (*p*‐values: 0.00047, 0.00027, 0.000036, and 0.000011, all < 0.05). These results quantitatively confirm that TGSP possesses intracellular free radical scavenging capabilities.

Flow cytometry analysis further quantitatively corroborated this result. The proportion of cells with high ROS levels was significantly reduced from 98.50% ± 1.10% in the H_2_O_2_‐treated group (*p* = 0.00023) to 80.10% ± 0.60%, 65.70% ± 1.30%, 50.00% ± 1.50%, and 41.20% ± 0.90% in the groups pre‐treated with TGSP (50, 100, 200, and 400 μg/mL), respectively (Figure 8B; *p*‐values: 0.00013, 0.00054, 0.000018, and 0.00036). These results indicate that TGSP can effectively scavenge excess intracellular ROS, thereby mitigating oxidative stress damage.

#### 3.9.2 TGSP Inhibits H_2_O_2_‐Induced Apoptosis

Flow cytometry analysis of Annexin V‐FITC/PI dual‐stained cells revealed that, compared to the normal control group (early apoptosis: 0.31% ± 0.06%; late apoptosis: 3.18% ± 0.36%), treatment with H_2_O_2_ (400 μM) significantly induced apoptosis in RAW264.7 cells (Figure 8C, *p* < 0.05). In the H_2_O_2_‐treated group, the proportion of early apoptotic cells increased to 4.55% ± 0.26% (*p* = 0.000027), and late apoptotic cells dramatically increased to 37.90% ± 1.30% (*p* = 0.00014), consequently leading to a corresponding decrease in the proportion of viable cells to 54.90% ± 2.43% (*p* = 0.00029).

However, pretreatment with TGSP effectively inhibited this apoptotic process in a dose‐dependent manner. At the highest concentration (400 μg/mL), TGSP significantly reduced the early apoptosis rate from 4.55% to 1.00% ± 0.12% (*p* = 0.00006) and the late apoptosis rate from 37.90% to 11.00% ± 0.28% (*p* = 0.0023), ultimately restoring the proportion of viable cells from 54.90% to 87.20% ± 1.86% (*p* = 0.00054).

#### 3.9.3 TGSP Suppresses Caspase‐3 and Caspase‐9 Activation

Consistent with the apoptosis rate results, TGSP significantly inhibited the H_2_O_2_‐induced activation of caspase‐3 and caspase‐9 (Figure 8D,E). The activities of these two apoptotic effector caspases were significantly elevated in H_2_O_2_‐treated cells. Compared to the control group, the protein levels in the model group increased from 9.772 ± 0.715 to 19.949 ± 1.023 U/mg protein for caspase‐3 (*p* = 0.0027) and from 8.963 ± 0.269 to 18.231 ± 1.092 U/mg protein for caspase‐9 (*p* = 0.003), respectively.

In contrast, these activities were dose‐dependently reduced in cells pre‐treated with TGSP, with the highest concentrations (200 and 400 μg/mL) showing the most significant inhibitory effects (*p* < 0.05). Caspase‐3 activity was reduced from 19.94 ± 1.02 U/mg protein in the H_2_O_2_ group to 11.11 ± 0.29 U/mg protein in the 400 μg/mL TGSP group (*p* = 0.0025). Similarly, caspase‐9 activity decreased from 18.23 ± 1.09 U/mg protein to 10.71 ± 0.79 U/mg protein in the 400 μg/mL TGSP group (*p* = 0.001).

## 4 Discussion

TGSP also inhibited H_2_O_2_‐induced cell apoptosis, as demonstrated by the reduced proportion of Annexin V‐FITC/PI double‐positive cells and the decreased activities of caspase‐3 and caspase‐9. These results suggest that TGSP may exert its protective effect by interfering with the mitochondria‐mediated intrinsic apoptosis pathway (Yuan and Ofengeim 2023). Oxidative stress can trigger changes in mitochondrial membrane permeability, leading to the release of cytochrome c and subsequent activation of the caspase cascade (Gao et al. 2017; Han et al. 2018; Jie et al. 2012). These findings provide molecular‐level evidence for TGSP as a potential cytoprotective agent.

### 4.1 Limitations of the Study

Although this study provides solid in vitro evidence for the biological activities of TGSP, it possesses several inherent limitations that need to be addressed in future research.

First, this study was based entirely on in vitro cell models and lacks in vivo validation. After oral administration, polyphenolic compounds undergo complex absorption, distribution, metabolism, and excretion (ADME) processes within the body, and their bioavailability is often low. Metabolism by the gut microbiota and the liver can convert them into metabolites with different, or even enhanced, activity. Therefore, in the absence of data from animal models, the actual in vivo efficacy, optimal dosage, potential toxicity, and true preventive or therapeutic effects of TGSP on complex diseases remain unknown. Establishing suitable animal models, such as an LPS‐induced acute inflammation model or an oxidative stress‐related disease model, is a crucial next step to validate the conclusions of this study.

Second, the investigation into the molecular mechanisms remains speculative. Although our results strongly point to the influence of TGSP on the NF‐κB, MAPK, and intrinsic apoptosis pathways, we did not directly measure the phosphorylation levels or nuclear translocation of key proteins within these pathways, such as p38, JNK, and NF‐κB p65. Future research should employ techniques like Western blotting to directly verify the activity changes of these core signaling molecules or utilize multi‐omics technologies such as transcriptomics and proteomics to more systematically uncover the molecular network through which TGSP regulates cellular functions.

Finally, from a methodological standpoint, this study failed to provide a voucher specimen for the plant material used. This, to some extent, affects the long‐term reproducibility of the research findings, as the chemical composition of plant materials can vary between different batches or sources. Future studies will strictly adhere to scientific protocols to ensure that all biological materials are supported by a corresponding voucher specimen for verification.

## 5 Conclusions

This study aimed to systematically evaluate the protective effect of TGSP on macrophages and to test the scientific hypothesis that it counteracts oxidative stress and inflammatory damage through multi‐target mechanisms. The research findings have clearly confirmed this hypothesis. We successfully prepared high‐purity TGSP, with a total polyphenol content of 76.37%, and identified that it is rich in key active components such as gallic acid, catechin, and epicatechin. At the cellular level, TGSP demonstrated potent and multi‐pathway protective activities:

- Anti‐inflammation: TGSP effectively inhibited the overproduction of key pro‐inflammatory mediators (NO, TNF‐α, and IL‐6) in LPS‐induced RAW264.7 cells.
- Antioxidation: TGSP not only directly scavenged various free radicals but also significantly mitigated H_2_O_2_‐induced oxidative damage by enhancing the activity of endogenous antioxidant enzymes (SOD, CAT, and GSH‐Px) and clearing excess intracellular ROS.
- Anti‐apoptosis: TGSP effectively blocked the apoptotic program triggered by oxidative stress by inhibiting the activities of the initiator caspase‐9 and the executioner caspase‐3, both of which are pivotal to the mitochondrial apoptosis pathway.

In conclusion, this study confirms that *T. grandis* seed polyphenol is an efficient cytoprotective agent with a multi‐targeted mechanism of action. This mechanism encompasses the direct inhibition of inflammatory responses, the activation of the endogenous antioxidant system, and the blockade of key cell apoptosis pathways. These findings provide a solid scientific basis for the advanced development of *T. grandis* , a traditional resource for both food and medicine. Furthermore, they indicate its great potential for application as a functional food ingredient or natural pharmaceutical component for the prevention and auxiliary intervention of various diseases related to oxidative stress and inflammation.

## Author Contributions

**Ran Liu:** conceptualization (lead), data curation (lead), formal analysis (lead), investigation (lead), methodology (lead), validation (lead), visualization (lead), writing – original draft (lead). **Baogang Zhou:** resources (supporting). **Kundian Che:** resources (supporting). **Wei Gao:** resources (supporting). **Haoyuan Luo:** resources (supporting). **Jialin Yang:** resources (supporting). **Zhanjun Chen:** resources (supporting). **Wenzhong Hu:** funding acquisition (lead), supervision (lead), writing – review and editing (supporting).

## Conflicts of Interest

The authors declare no conflicts of interest.

## Supporting information


Data S1

## Data Availability

The original contributions presented in this study are included in the article/Data S1; further inquiries can be directed to the corresponding author.
