# Supplementary material for: Torreya grandis Seed Polyphenols Protect RAW264.7 Macrophages by Inhibiting Oxidative Stress and Inflammation
Source: Food Sci Nutr. 2025 Jul 29;13(8):e70682. doi: 10.1002/fsn3.70682 (PMC12305672; doi:10.1002/fsn3.70682)
Supplement: Supplementary file 1 — Data S1 [file FSN3-13-e70682-s001.docx]

**Supporting Information**

***Torreya grandis* Seed Kernel Polyphenols Protect RAW264.7 Macrophages by Inhibiting Oxidative Stress and Inflammation**

**S1 Optimization of Ultrasound-Assisted Extraction Conditions**

**S1.1 Single-Factor Experiments**

The extraction yield of polyphenols from *T. grandis* kernels was selected as the evaluation index. Single-factor experiments were conducted to systematically investigate the effects of ultrasonic power, sonication time, solid-to-liquid ratio, ethanol concentration, and ultrasonic temperature on the extraction efficiency. During each experiment, one factor was varied while maintaining other parameters at fixed levels. The optimization intervals for each factor were designed as follows:

a) Ultrasonic Power The influence of ultrasonic power (150, 180, 210, 240, and 270 W) on polyphenol extraction yield was investigated while maintaining other parameters constant: sonication time 50 min, solid-to-liquid ratio 1:30, ethanol concentration 70%, and ultrasonic temperature 55 °C.

b) Sonication Time The effect of sonication time (30, 40, 50, 60, and 70 min) was examined with fixed parameters: ultrasonic power 210 W, solid-to-liquid ratio 1:30, ethanol concentration 70%, and ultrasonic temperature 55 °C.

c) Solid-to-Liquid Ratio The impact of solid-to-liquid ratio (1:10, 1:20, 1:30, 1:40, and 1:50) was studied while maintaining: ultrasonic power 210 W, sonication time 50 min, ethanol concentration 70%, and ultrasonic temperature 55 °C.

d) Ethanol Concentration The influence of ethanol concentration (60%, 65%, 70%, 75%, and 80%) was investigated under fixed conditions: ultrasonic power 210 W, sonication time 50 min, solid-to-liquid ratio 1:30, and ultrasonic temperature 55 °C.

e) Ultrasonic Temperature The effect of ultrasonic temperature (45, 50, 55, 60, and 65 °C) was examined with other parameters held constant: ultrasonic power 210 W, sonication time 50 min, solid-to-liquid ratio 1:30, and ethanol concentration 70%.

All experiments were performed in triplicate, and mean values were calculated. Total polyphenol content in the extracts was determined using the Folin-Ciocalteu colorimetric method. The results of these single-factor experiments established suitable ranges for each parameter, providing a foundation for subsequent response surface optimization experiments.

**S1.2 Response Surface Methodology Optimization Design**

Based on the results of single-factor experiments, a Box-Behnken design (BBD) was employed to optimize the extraction process of polyphenols from *T. grandis* kernels. The optimization incorporated three independent variables: sonication time (A), liquid-to-solid ratio (B), and ethanol concentration (C), with polyphenol extraction yield (Y) as the response value. A three-factor, three-level response surface experimental design was implemented.

The levels for each factor were determined based on the optimal values identified in the single-factor experiments and their adjacent values. The coded levels of the factors are presented in Table S1.

Table S1. Design of factors and levels for response surface methodology

| Level | Ultrasonic time  (A)/min | Liquid-solid ratio (B)/mL/g | Ethanol concentration (C)/% |
| --- | --- | --- | --- |
| -1 | 40 | 30 | 65 |
| 0 | 50 | 40 | 70 |
| 1 | 60 | 50 | 75 |

**S2 Results**

**S2.1 Analysis of Single-Factor Experimental Results**

**S2.1.1 Effect of Ultrasonic Power**

The influence of ultrasonic power on polyphenol extraction yield from *T. grandis* kernels within the range of 150-270 W is illustrated in Figure S1A. The extraction yield demonstrated an initial increase followed by stabilization as power increased. In the range of 150-210 W, the extraction yield significantly increased from 6.592 mg/g to 7.507 mg/g, primarily attributed to enhanced cavitation effects promoting cell wall disruption. When the power was further increased to 240-270 W, no significant change in extraction yield was observed, indicating that excessive power might result in energy waste. Considering both extraction efficiency and operational stability, 210 W was determined as the optimal ultrasonic power(Yan et al. 2023).

**S2.1.2** Effect of Ultrasonic Time

The influence of ultrasonic time on extraction yield exhibited a significant "inverted U-shaped" trend (Figure S1B). As the duration increased from 30 min to 50 min, the extraction yield rose from 6.724 mg/g to 8.082 mg/g. However, further extension of time led to a significant decrease in yield, possibly due to polyphenol degradation caused by prolonged ultrasonic exposure. The minimum standard deviation (0.098) was observed at 50 min, indicating optimal experimental reproducibility; therefore, 50 min was determined as the optimal ultrasonic duration(Wen et al. 2022).

**S2.1.3** Effect of Liquid-Solid Ratio

As shown in Figure S1C, the extraction yield increased significantly as the liquid-solid ratio increased from 1:10 to 1:40, primarily due to enhanced polyphenol dissolution driven by a larger concentration gradient. The maximum yield (7.697 mg/g) was achieved at 1:40, while further increases in the ratio resulted in a slight decline in extraction yield. Considering solvent consumption and extraction efficiency, 1:40 was selected as the optimal liquid-solid ratio(Hu et al. 2022).

**S2.1.4** Effect of Ethanol Concentration

Ethanol concentration significantly influenced extraction efficiency (Figure S1D). When the concentration increased from 60% to 70%, the extraction yield rose from 4.743 mg/g to a maximum of 7.962 mg/g, likely due to the optimal solvent polarity for target polyphenol dissolution. Further increases in ethanol concentration led to a significant decrease in extraction yield, indicating 70% as the optimal ethanol concentration(Zhu et al. 2024).

**S2.1.5** Effect of Ultrasonic Temperature

Within the temperature range of 45-55 ℃, the extraction yield increased with rising temperature (Figure S1E), attributable to enhanced solvent penetration and polyphenol dissolution at elevated temperatures. However, when the temperature exceeded 55 ℃, a significant decrease in extraction yield was observed, presumably due to the degradation of thermally sensitive polyphenols. Therefore, 55 ℃ was determined as the optimal ultrasonic temperature(Zhang et al. 2020).

Through single-factor experiments, the suitable ranges of process parameters were preliminarily established, laying the foundation for subsequent response surface optimization. The results indicated that the optimal process conditions for ultrasound-assisted extraction of polyphenols from *T. grandis* kernels were: ultrasonic power of 210 W, ultrasonic duration of 50 min, liquid-solid ratio of 1:40, ethanol concentration of 70 %, and ultrasonic temperature of 55 ℃.


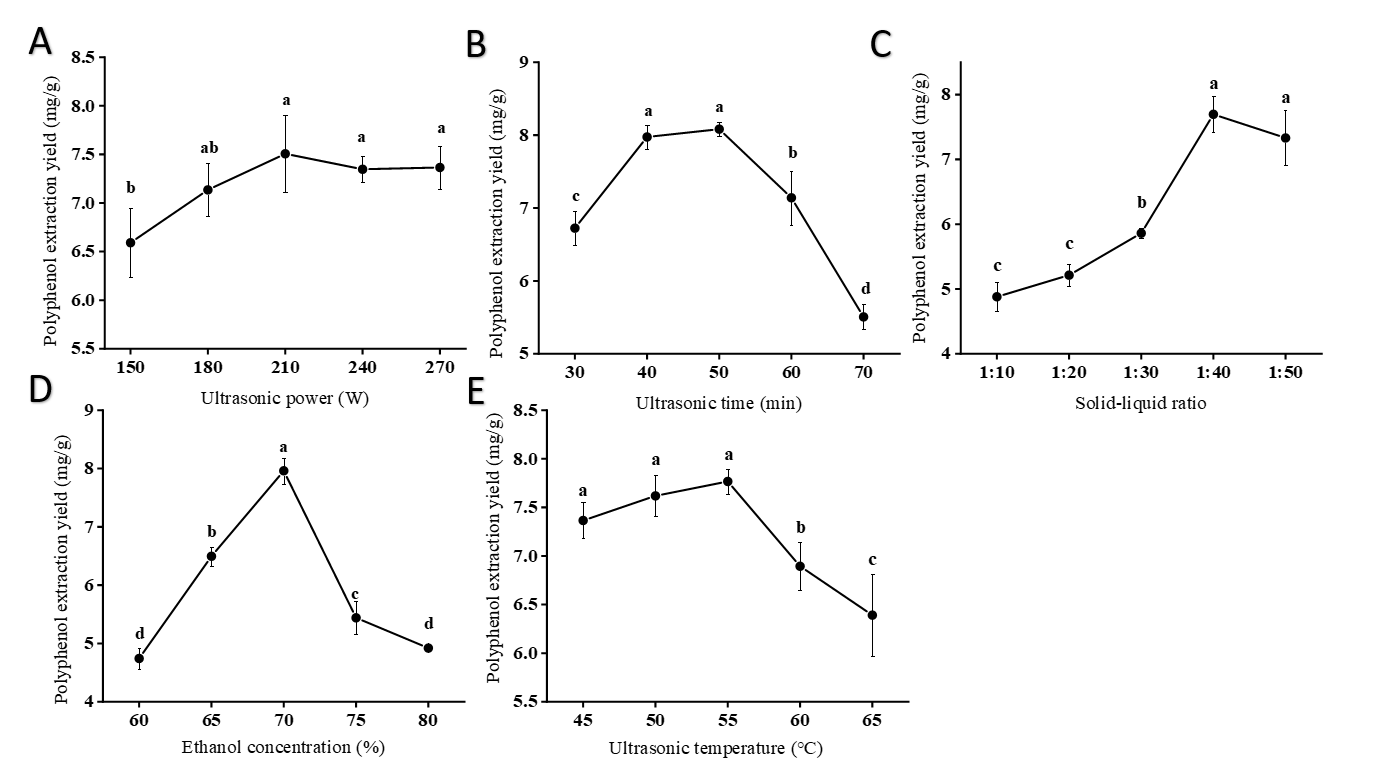


Figure S1. Effects of ultrasonic extraction parameters on polyphenol yield from *T. grandis* seeds. (A) ultrasonic power, (B) ultrasonic time, (C) liquid-solid ratio, (D) ethanol concentration, and (E) ultrasonic temperature. Different letters above the bars indicate significant differences (*p* < 0.05). Error bars represent standard deviation (n = 3).

**S2.2 Analysis of Response Surface Optimization Results**

**S2.2.1** Establishment and Significance Analysis of Response Surface Model

Based on data obtained from the Box-Behnken experimental design, a quadratic polynomial regression equation was established through multiple regression analysis to describe the relationship between polyphenol extraction yield (Y) from *T. grandis* kernels and three process parameters:

Y = 8.74 + 0.0925X₁ + 0.1213X₂ + 0.1363X₃ - 0.005X₁X₂ - 0.09X₁X₃ - 0.0125X₂X₃ - 0.6368X₁² - 0.2693X₂² - 0.4392X₃²

where X₁, X₂, and X₃ represent the coded values of ultrasonic time, liquid-solid ratio, and ethanol concentration, respectively. Analysis of variance demonstrated the model's statistical significance (F = 82.72, P < 0.0001), while the lack of fit was not significant (P = 0.4047 > 0.05), indicating that the model accurately describes the relationship between process parameters and response values.

The model's coefficient of determination (R²) was 0.9907, indicating that 99.07% of experimental data variation could be explained by the model. The difference between the adjusted R² value (0.9787) and predicted R² value (0.9206) was less than 0.2, demonstrating the model's strong predictive capability. Furthermore, the coefficient of variation was merely 0.8434%, well below the acceptable standard of 10%, confirming both the reliability of experimental data and the model's precision.

The positive coefficients of the linear terms in the regression equation indicated that all three process parameters had positive effects on extraction yield, with their influence ranking as follows: ethanol concentration (0.1363) > liquid-solid ratio (0.1213) > ultrasonic time (0.0925). The negative quadratic coefficients suggested the existence of optimal points between process parameters and response values. These findings provided a reliable mathematical foundation for subsequent optimization.

Table S2. Response surface experimental design and results

| Number | Ultrasonic time (A)/min | Liquid-solid ratio (B)/mL/g | Ethanol concentration (C)/% | Polyphenol extraction yield (mg/g) |
| --- | --- | --- | --- | --- |
| 1 | 40 | 30 | 70 | 7.55 |
| 2 | 60 | 30 | 70 | 7.82 |
| 3 | 40 | 50 | 70 | 7.85 |
| 4 | 60 | 50 | 70 | 8.10 |
| 5 | 40 | 40 | 65 | 7.38 |
| 6 | 60 | 40 | 65 | 7.67 |
| 7 | 40 | 40 | 75 | 7.83 |
| 8 | 60 | 40 | 75 | 7.76 |
| 9 | 50 | 30 | 65 | 7.78 |
| 10 | 50 | 50 | 65 | 8.00 |
| 11 | 50 | 30 | 75 | 8.08 |
| 12 | 50 | 50 | 75 | 8.25 |
| 13 | 50 | 40 | 70 | 8.79 |
| 14 | 50 | 40 | 70 | 8.71 |
| 15 | 50 | 40 | 70 | 8.68 |
| 16 | 50 | 40 | 70 | 8.68 |
| 17 | 50 | 40 | 75 | 8.82 |

Table S3. Analysis of variance for response surface regression model

| Source of variation | Sum of squares | DF | Mean square | F-value | P-value | Significance |
| --- | --- | --- | --- | --- | --- | --- |
| Model | 3.48 | 9 | 0.3863 | 82.72 | < 0.0001 | ** |
| A-Ultrasonic time | 0.0685 | 1 | 0.0685 | 14.66 | 0.0065 | ** |
| B-Liquid ratio | 0.1176 | 1 | 0.1176 | 25.18 | 0.0015 | ** |
| C-Ethanol concentration | 0.1485 | 1 | 0.1485 | 31.80 | 0.0008 | ** |
| AB | 0.0001 | 1 | 0.0001 | 0.0214 | 0.8878 | * |
| AC | 0.0324 | 1 | 0.0324 | 6.94 | 0.0337 | ** |
| BC | 0.0006 | 1 | 0.0006 | 0.1338 | 0.7253 | * |
| A² | 1.71 | 1 | 1.71 | 365.50 | < 0.0001 | ** |
| B² | 0.3052 | 1 | 0.3052 | 65.35 | < 0.0001 | ** |
| C² | 0.8124 | 1 | 0.8124 | 173.93 | < 0.0001 | ** |
| Residual | 0.0327 | 7 | 0.0047 |  |  |  |
| Lack of fit | 0.0158 | 3 | 0.0053 | 1.24 | 0.4047 | N |
| Pure error | 0.0169 | 4 | 0.0042 |  |  |  |
| Total | 3.51 | 16 |  |  |  |  |

DF = Degrees of freedom; ** indicates highly significant; * indicates significant; N indicates not significant

**S2.2.2** Analysis of Interactive Effects Between Process Parameters

The response surface analysis revealed complex interactions among ultrasonic extraction parameters. The contour plots and response surface graphs in Figure S2 illustrated the interactive characteristics between three pairs of process parameters.

The interaction between ultrasonic time (X₁) and liquid-solid ratio (X₂) exhibited relatively independent characteristics. The contour plot displayed regular elliptical patterns, with a small interaction coefficient (-0.005) and a P-value of 0.8878, significantly exceeding the 0.05 significance level. This independence suggested that these parameters could be optimized separately, thereby simplifying the process optimization complexity.

A significant interaction was observed between ultrasonic time (X₁) and ethanol concentration (X₃), visually evident from the distorted contour lines. The interaction coefficient of -0.09 and P-value of 0.0337 indicated that their combined effect significantly influenced extraction efficiency. Specifically, at lower ethanol concentrations, extended ultrasonic duration substantially increased extraction yield, whereas at higher ethanol concentrations, prolonged ultrasonic exposure actually reduced extraction efficiency. This interactive characteristic emphasized the necessity of considering their synergistic effects during process optimization.

The interaction between liquid-solid ratio (X₂) and ethanol concentration (X₃) demonstrated relatively weak coupling, maintaining relatively regular contour patterns. The interaction coefficient was merely -0.0125 with a P-value of 0.7253, indicating non-significant interaction effects. This finding simplified the parameter optimization process, suggesting that these parameters could be adjusted independently within certain ranges.

The analysis of parameter interactions not only deepened the understanding of the extraction process but also provided theoretical foundations for precise parameter control. The significant interaction between ultrasonic time and ethanol concentration, in particular, warrants special attention in practical production to achieve optimal extraction conditions.


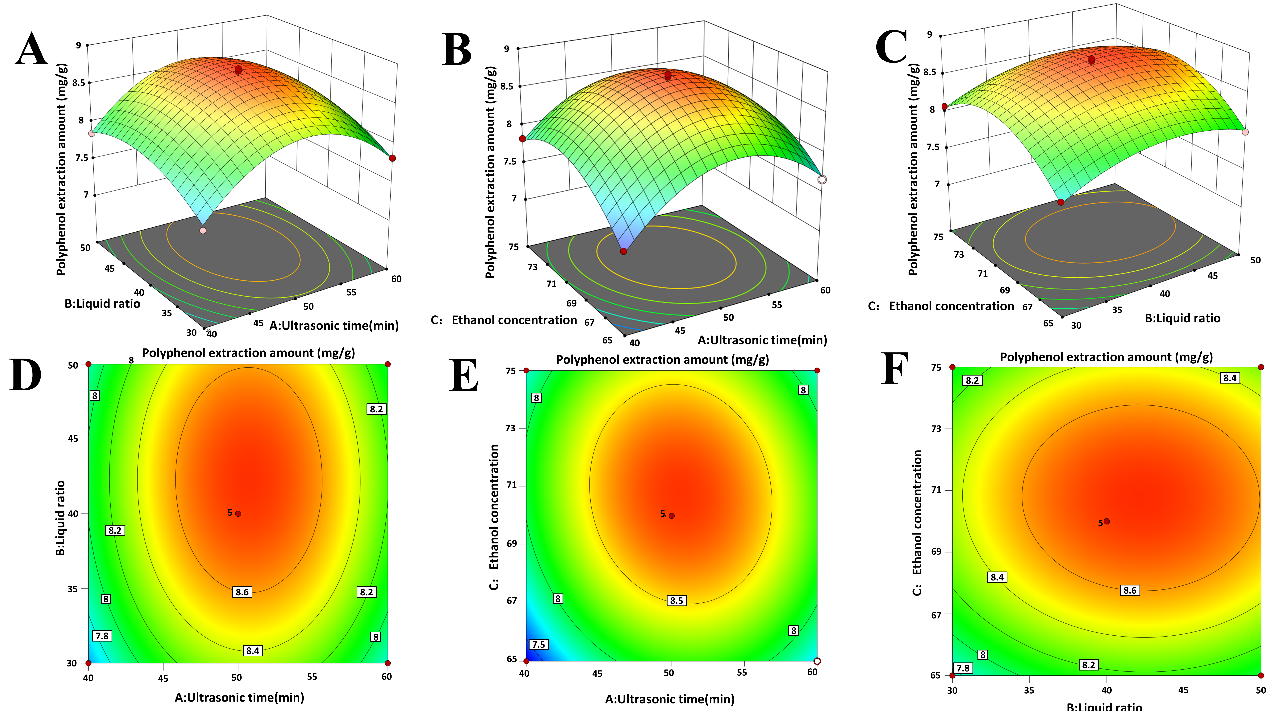


Figure S2. Response surface plots (3D) and contour plots (2D) illustrating the interactive effects of extraction parameters on the polyphenol yield from Torreya grandis kernels. The interactions shown are between: (A, D) ultrasonic time and liquid-to-solid ratio; (B, E) ultrasonic time and ethanol concentration; and (C, F) liquid-to-solid ratio and ethanol concentration. When analyzing the interaction between two variables, the third variable was held constant at its central level (0). The color gradient in the contour plots represents the polyphenol yield (mg/g).

**S2.2.3** Optimization and Verification of Process Parameters

Through numerical optimization of response surface analysis, the optimal process conditions for polyphenol extraction from *T. grandis* kernels were determined: ultrasonic duration of 50.615 min, liquid-solid ratio of 42.205 mL/g, and ethanol concentration of 70.728%, while maintaining ultrasonic power at 210 W and ultrasonic temperature at 55 ℃. Under these optimized conditions, the model predicted a polyphenol extraction yield of 8.762 mg/g. For practical convenience, the optimized process parameters were slightly modified and set as follows: an ultrasonic power of 210 W, a temperature of 55 °C, a time of 50 min, a solid-to-liquid ratio of 1:40 g/mL, and an ethanol concentration of 70%.

To verify the model's accuracy and reliability, three parallel validation experiments were conducted. The experimental results yielded polyphenol extraction values of 8.789, 8.748, and 8.856 mg/g, with a mean value of 8.797 ± 0.055 mg/g (n = 3). The relative standard deviation (RSD) of experimental data was 0.63%, demonstrating excellent process reproducibility. The relative error between the experimental mean and model prediction was merely 0.40%, comprehensively validating the response surface model's outstanding predictive accuracy.

**References**

Hu, Y., Suo, J., Jiang, G., Shen, J., Cheng, H., Lou, H., Yu, W., Wu, J., and Song, L. 2022. "The effect of ethylene on squalene and beta-sitosterol biosynthesis and its key gene network analysis in *T. grandis*nuts during post-ripening process." *Food Chem*, *368*, 130819. <https://doi.org/10.1016/j.foodchem.2021.130819>

Wen, S., Lu, Y., Yu, N., Nie, X., and Meng, X. 2022. "Microwave pre-treatment aqueous enzymatic extraction (MPAEE): A case study on the *T. grandis*seed kernels oil." *Journal of Food Processing and Preservation*, *46*(11), Article e17115. <https://doi.org/10.1111/jfpp.17115>

Yan, J., Zeng, H., Chen, W., Zheng, S., Luo, J., Jiang, H., Yang, B., Farag, M. A., Lou, H., Song, L., and Wu, J. 2023. "Effects of tree age on flavonoids and antioxidant activity in *T. grandis*nuts via integrated metabolome and transcriptome analyses." *Food Frontiers*, *4*(1), 358-367. <https://doi.org/10.1002/fft2.211>

Zhang, Z. Y., Jin, H. B., Suo, J. W., Yu, W. Y., Zhou, M. Y., Dai, W. S., Song, L. L., Hu, Y. Y., and Wu, J. S. 2020. "Effect of Temperature and Humidity on Oil Quality of Harvested *T. grandis*cv. Merrillii Nuts During the After-Ripening Stage." *Frontiers in Plant Science*, *11*, Article 573681. <https://doi.org/10.3389/fpls.2020.573681>

Zhu, R., Gao, N., Luo, J., and Shi, W. 2024. "Genome and Transcriptome Analysis of the *T. grandis*WRKY Gene Family during Seed Development." *Genes (Basel)*, *15*(3). <https://doi.org/10.3390/genes15030267>
